# Cross-Omic Comparative Analysis Identifies Transcriptomic Signatures and Exploratory Gene Set-Level Signals in Sporadic Creutzfeldt–Jakob Disease

**DOI:** 10.3390/ijms27156560

**Published:** 2026-07-23

**Authors:** Ramandeep Kaur Oberoi, Deepti Gurung, Laura K. Harris

**Affiliations:** 1 Department of Health Informatics, School of Health Professions, Rutgers, The State University of New Jersey, Newark, NJ 07107, USA; ro352@shp.rutgers.edu; 2 Harris Analytics Institute, DeWitt, MI 48820, USA; deepti.gurung@harrisai.org

**Keywords:** sporadic Creutzfeldt–Jakob disease, prion disease, cross-omic analysis, gene set enrichment, cell–substrate adhesion, DNA methylation, transcriptomics, miRNA

## Abstract

Sporadic Creutzfeldt–Jakob disease (sCJD) is a rapidly progressive fatal neurodegenerative disease whose basis of pathogenesis remains incompletely defined. Public datasets allow molecular changes to be compared across transcriptomic, post-transcriptional, and epigenomic layers, but comparisons are limited by differences in tissue source and platform. We performed a cross-omic comparative analysis of four sCJD case–control datasets spanning mRNA, miRNA, and DNA methylation layers. In this study, cross-omic refers to comparison of independently analyzed molecular datasets rather than joint multi-omic modeling of matched molecular layers from the same samples. Differential feature analysis, overrepresentation analysis, and gene set enrichment analysis were conducted within each layer. Reproducibility was evaluated across discovery and validation cohorts, followed by cross-layer comparison at the gene level and GO-BP gene set level. FDR-controlled overlap was treated as the primary analysis, whereas nominal *p* < 0.05 overlap was considered exploratory. Gene-level overlap across molecular layers was limited, consistent with tissue and platform heterogeneity. Under FDR-controlled criteria, cross-omic GO-BP gene set overlap was not observed. Exploratory nominal-threshold analyses identified recurrent gene set-level signals, mainly between transcriptomic and DNA methylation datasets, including GO-BP terms related to cell–substrate adhesion. These findings support cross-omic comparative analysis as a framework for identifying hypothesis-generating patterns in rare neurodegenerative diseases.

## 1. Introduction

Creutzfeldt–Jakob disease (CJD) is the most prevalent human prion disease [1,2,3]. CJD is a rapidly progressive, ultimately fatal neurodegenerative disorder that includes sporadic CJD (sCJD; 85%), genetic (gCJD; 10–15%), iatrogenic (iCJD; <1%), and variant CJD (vCJD; <1%). sCJD predominates, at approximately 1–2 cases per million per year [3,4]. Clinically, sCJD manifests as spongiform encephalopathy characterized by rapidly progressing dementia and ultimately death, with a median survival of about 4–6 months, and 70% of patients die within one year of disease onset [4,5,6]. The median age at onset or death in sCJD is approximately 67–68 years, and the majority of cases occur between the ages of about 55 and 80 [7,8].

CJD is widely characterized as a proteinopathy, developing when the normal prion protein, abundantly expressed in neurons, undergoes conformational misfolding into its pathogenic β-sheet-rich form [9]. This infectious protein can template the conformational conversion of normal cellular prion protein (PrP^C), promoting a self-perpetuating misfolding process that spreads through neural tissue [10]. However, accumulating evidence suggests that additional mechanisms, including broader protein misfolding pathways and potential cross-seeding interactions, may also contribute to neurodegenerative pathology [11]. Research continues to elucidate how prions acquire infectivity de novo, with evidence suggesting a role for rare pathogenic mutations in the PRNP gene (encoding normal prion protein) on chromosome 20 and the codon 129 polymorphism of PRNP (methionine or valine) [12,13]. Individuals homozygous at this site (MM or VV) are at increased risk of disease compared to heterozygous (MV) [14]. Additionally, a genome-wide association study of sCJD revealed susceptibility loci at STX6 and GAL3ST1, with additional signals at PDIA4 and nearby BMERB1 approaching genome-wide significance [15,16]. Despite decades of research, the molecular basis of disease onset and progression remains incompletely understood, and no effective disease-modifying therapies are currently available.

Several single-omics studies have characterized individual molecular layers in sCJD, including genetic profiling, mRNA transcriptomic profiling, miRNA profiling, and genome-wide DNA methylation [17,18,19]. However, cross-omics comparative analysis across these layers remains limited by dataset availability, cohort heterogeneity, and the lack of matched multi-omic samples. As a result, it remains unclear to what extent molecular signals are reproducible across layers or whether consistent biological processes can be revealed across independent datasets.

Analyzing transcriptomic, miRNA, and DNA methylation datasets in parallel provides an opportunity to evaluate complementary layers of gene regulation, allowing comparison of epigenetic and post-transcriptional variation alongside observed changes in gene expression. Rather than assuming unified multi-omic integration, this approach enables assessment of whether convergence occurs at the level of individual genes or, more robustly, at the level of biological processes.

Here, we present a cross-omic analysis of published microarray, RNA-seq, miRNA-seq, and DNA methylation datasets from frontal cortex and whole-blood samples of sCJD patients and healthy controls. We use “cross-omic” to refer to the comparison of independently analyzed transcriptomic, miRNA, and DNA methylation datasets, rather than joint multi-omic modeling of matched molecular layers from the same individuals. We first revealed differentially expressed features (DEFs), which were used for overrepresentation analysis using Fisher’s exact test (FET). In parallel, gene set enrichment analysis (GSEA) was performed using pre-ranked gene lists derived from the full dataset. Predictive models were also constructed to evaluate the discriminatory capacity of molecular signatures. For miRNA and DNA methylation datasets, models were trained and tested using internal discovery and validation cohorts generated by a 75/25 split, while transcriptomic datasets were evaluated across independent discovery and validation cohorts. Logistic regression and random forest models were used to classify sCJD and control samples based on discovery-derived molecular signatures. Logistic regression was assessed using leave-one-out cross-validation (LOOCV), and random forest using repeated stratified K-fold cross-validation.

In summary, this cross-omic analysis revealed exploratory recurrence of GO-BP terms related to cell–substrate adhesion across transcriptomic and DNA methylation datasets in sCJD. The recurrence of this signal across brain- and blood-derived datasets supports its potential biological relevance, while also requiring cautious interpretation because the datasets differ in tissue source and molecular platform. More broadly, these results suggest that GO-BP gene set-level signals may provide a more stable representation of disease-associated molecular alterations than individual features, highlighting the value of cross-omic comparison for identifying consistent biological processes in sCJD.

## 2. Results

### 2.1. Transcriptomic Profiling in Brain Reveals Immune Activation and Synaptic Suppression in sCJD

To identify genes and gene sets associated with sCJD in the transcriptomic domain, GSE124571 and GSE214373 were used as discovery and validation datasets, respectively. Differential expression signatures were first derived from the discovery dataset by comparing sCJD samples with non-neurological controls. Genes that met statistical thresholds were subsequently evaluated in the independent validation cohort to assess reproducibility across platforms. Gene set-level analyses, including overrepresentation testing (FET) and GSEA, were then performed to determine whether transcriptional alterations were associated with shared biological processes.

#### 2.1.1. Widespread Differential Gene Expression Is Reproducible Across Independent Brain Cohorts

**Figure 1:**
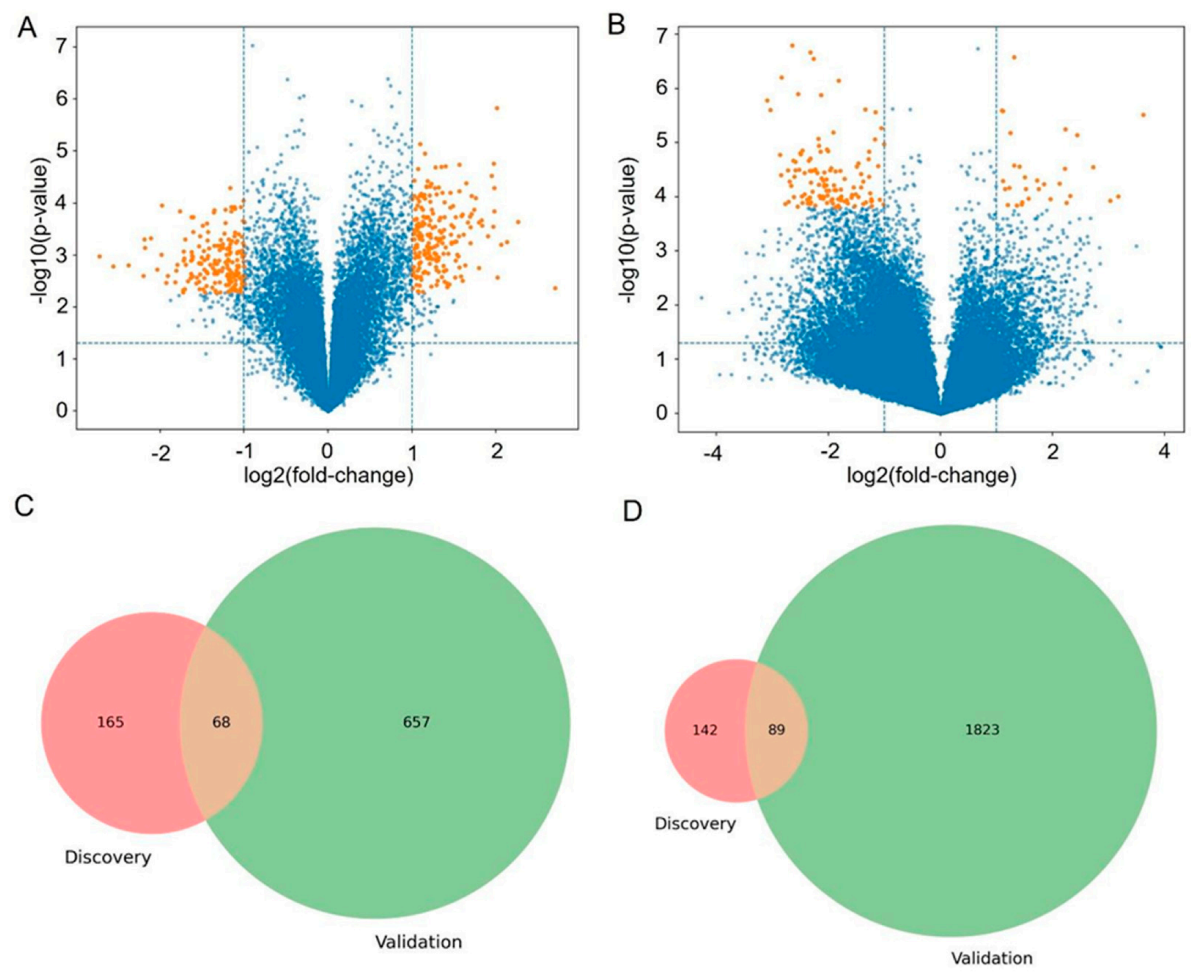
Reproducibility of differential gene expression across discovery and validation transcriptomic cohorts. (**A**) Volcano plot of differential expression in the discovery dataset (GSE124571). Each point represents a probe plotted by log2(fold change) versus –log10(*p*-value). (**B**) Volcano plot of differential expression in the validation dataset (GSE214373), displayed using the same thresholds as the discovery dataset. The validation dataset showed a more pronounced skew toward under-expressed features than the discovery dataset. (**C**) Venn diagram showing overlap of nominally significant (*p* < 0.05) over-expressed gene-symbols between discovery and validation datasets. (**D**) Venn diagram showing overlap of nominally significant (*p* < 0.05) under-expressed symbols between discovery and validation datasets. In panels (**A**,**B**), vertical dashed lines indicate the fold-change cutoff (|log2FC| = 1), the horizontal dashed line indicates the nominal significance threshold (*p* = 0.05), and orange points indicate probes with FDR < 0.05. In panels (**C**,**D**), red circles represent the discovery cohort, green circles represent the validation cohort, and overlapping regions indicate shared gene symbols between cohorts.

Differential expression analysis of the discovery dataset, comprising 24,525 probes (e.g., ILMN_2135258), revealed 498 probes as differentially expressed using nominal Welch’s *t*-test *p* < 0.05 and |log2FC| > 1 thresholds, including 244 over-expressed and 254 under-expressed probes (Table S1). However, only 398 probes (213 over- and 185 under-expressed) remained significant after multiple-testing correction (Benjamini–Hochberg false discovery rate correction; FDR-BH < 0.05). A volcano plot illustrates the distribution of these probes, with over-expressed probes (positive log2(fold change)) located in the upper-right region and under-expressed probes (negative log2(fold change)) in the upper-left region, while probes with minimal expression change cluster centrally (Figure 1A). Probes occupying the extreme upper regions of the plot represent the most statistically robust disease-associated probes.

In the validation dataset, comprising 61,906 Ensembl gene IDs (e.g., ENSG00000279928), differential expression analysis revealed 3937 IDs at *p* < 0.05 with 885 IDs being over- and 3052 under-expressed (Table S2). Of these, 152 IDs remained significant following FDR-BH correction with 31 over- and 121 under-expressed IDs, indicating a stronger bias toward under-expression relative to discovery. The corresponding volcano plot (Figure 1B) supports this pattern, showing a more pronounced under-expression skew rather than a distribution directly comparable to the discovery dataset. These results indicate differential expression in affected brain tissue while also highlighting cohort- and/or platform-specific differences between the discovery and validation datasets.

To evaluate reproducibility across datasets, cross-cohort overlap analysis was performed on differentially expressed gene-symbols revealed using a nominal *p*-value threshold (*p* < 0.05) for concordant directionality (i.e., mRNAs classified as over-expressed or under-expressed in both cohorts). First, probes and IDs needed to be converted to gene symbols and duplicates were removed. This resulted in 464 symbols (233 over- and 231 under-expressed) remaining in the *p* < 0.05 group for the discovery dataset, and 371 symbols (203 over- and 168 under-expressed) in the FDR-BH group. For the validation dataset, 2637 symbols (725 over- and 1912 under-expressed) remained in the *p* < 0.05 group, while 100 symbols (27 over- and 73 under-expressed) were left in the FDR-BH group. For over-expressed symbols with *p* < 0.05, 165 were significant only in the discovery dataset, 657 were significant only in the validation dataset, and 68 were shared between cohorts (Figure 1C). For under-expressed symbols with *p* < 0.05, 142 were significant only in the discovery dataset, 1823 were significant only in the validation dataset, and 89 were shared between datasets (Figure 1D). Table 1 lists the shared symbols between discovery and validation datasets representing differential expression (*p* < 0.05) in mRNA associated with sCJD. Together, these results demonstrate widespread transcriptional dysregulation in sCJD and identify a subset of gene-level changes that reproducibly distinguish disease and control samples across independent brain cohorts.

To determine whether transcriptional signatures derived from the discovery dataset could discriminate disease status in an independent cohort, dimensionality reduction and classification analyses were performed using the validation dataset. PCA was conducted using DEG subsets defined in the discovery dataset (Figure S1). Both over- and under-expressed gene subsets demonstrated partial separation between sCJD and control samples along the first two principal components. The first two components together explained the majority of variance (over-expressed genes: PC1 = 53.3%, PC2 = 13.9%; under-expressed genes: PC1 = 76.6%, PC2 = 10.0%). The separation between groups was more pronounced when using under-expressed gene subsets, suggesting that transcriptional suppression contributes substantially to disease-associated variance. As an exploratory assessment of sample-level discrimination, classification performance was evaluated using logistic regression with LOOCV in the validation cohort. Models based on over-expressed genes showed slightly lower discrimination (AUC ROC = 0.889) whereas models based on under-expressed genes showed the strongest exploratory discrimination (AUC ROC = 0.956, Figure S2). Random forest classifiers using repeated stratified k-fold validation produced comparable performance (AUC ROC = 0.933 for over-expressed genes and AUC ROC = 0.911 for under-expressed genes; Figure S3). Additional operating-point and threshold-independent performance metrics, including sensitivity, specificity, precision, F1-score, PR-AUC, and AUC ROC, are provided in Table S3 and were interpreted descriptively because of the small validation cohort size.

#### 2.1.2. Gene Set Enrichment Highlights Immune-Associated Activation and Synaptic Suppression in Bulk Cortical Tissue

To determine whether gene-level transcriptional alterations were associated with coherent biological processes, gene set enrichment analyses were conducted in both the discovery and validation cohorts using complementary approaches: overrepresentation analysis by FET and GSEA. These methods capture complementary aspects of gene set enrichment, with FET identifying GO-BP categories overrepresented among differentially expressed genes and GSEA detecting coordinated shifts across the ranked transcriptome. Nominal *p* < 0.05 cross-cohort analyses are reported as exploratory sensitivity analyses, while FDR-adjusted results are retained as the primary multiple-testing-controlled results.

In the discovery cohort, FET revealed 748 up-regulated and 470 down-regulated GO-BP gene sets at nominal significance (*p* < 0.05), of which 405 up-regulated and 208 down-regulated gene sets remained significant after FDR correction (Table S4). Among up-regulated gene sets, the most significant GO-BP terms included positive regulation of the multicellular organismal process (OR = 3.19, FDR = 4.17 × 10^−8^) and regulation of the immune system process (OR = 2.97, FDR = 4.28 × 10^−7^), supported by overlapping genes including APOE, AIF1, CD14, CD68, C1QB, and C1QC. Additional highly enriched terms included lymphocyte activation and response to wounding, further supporting immune activation. In contrast, down-regulated gene sets were dominated by neuronal and synaptic processes, including synaptic signaling (OR = 8.02, FDR = 2.08 × 10^−25^), cell–cell signaling (OR = 5.24, FDR = 1.03 × 10^−18^), and vesicle-mediated transport in synapse (OR = 9.96, FDR = 1.69 × 10^−14^), with overlapping genes including ADCY1, AMPH, CADPS2, CPLX1, CPLX2, DNM1, and SNAP25. Together, these FET results indicate upregulation of immune-associated processes alongside suppression of synaptic and neuronal signaling functions in the discovery cohort.

GSEA of the ranked discovery transcriptome showed the same broad pattern. In the discovery cohort, GSEA revealed 1153 up-regulated and 326 down-regulated GO-BP gene sets at nominal significance (*p* < 0.05), of which 643 up-regulated and 83 down-regulated gene sets remained significant after FDR correction (Table S5). The strongest positively enriched GO-BP terms included cell adhesion mediated by integrin (NES = 2.47, FDR < 0.001), integrin-mediated signaling pathway (NES = 2.47, FDR < 0.001), and adaptive immune response (NES = 2.44, FDR < 0.001), followed by myeloid cell activation involved in immune response, cellular response to biotic stimulus, and lipopolysaccharide-mediated signaling. Conversely, the strongest negatively enriched gene sets were synaptic vesicle priming (NES = −2.48, FDR < 0.001), vesicle-mediated transport in synapse (NES = −2.40, FDR < 0.001), synaptic vesicle maturation (NES = −2.40, FDR < 0.001), and synaptic vesicle exocytosis (NES = −2.32, FDR < 0.001). Additional negatively enriched gene sets included neurotransmitter secretion (NES = −2.27, FDR < 0.001), presynaptic endocytosis (NES = −2.26, FDR < 0.001), and modulation of excitatory postsynaptic potential (NES = −2.28, FDR < 0.001). Thus, both FET and GSEA in the discovery cohort are consistent with coordinated immune activation together with suppression of synaptic transmission and neuronal communication.

Since these transcriptomic datasets were derived from bulk post-mortem frontal cortices, the observed immune-associated up-regulation and synaptic or neuronal down-regulation should be interpreted as tissue-level signatures. These patterns may reflect changes in cellular composition, including neuronal loss and reactive gliosis, as well as possible cell-intrinsic transcriptional regulation. The present analysis does not distinguish between these possibilities.

Broad thematic reproducibility was observed in the validation cohort, although the leading gene sets were not identical to those in discovery. FET revealed 1126 up-regulated and 410 down-regulated GO-BP gene sets at nominal significance (*p* < 0.05), with 504 up-regulated and 53 down-regulated gene sets remaining significant after FDR correction (Table S6). The strongest up-regulated gene sets in validation were positive regulation of the developmental process (OR = 2.75, FDR = 7.68 × 10^−11^), regulation of cell differentiation (OR = 2.59, FDR = 7.68 × 10^−11^), cell adhesion (OR = 2.60, FDR = 1.33 × 10^−10^), and cell activation (OR = 2.75, FDR = 9.64 × 10^−10^), while immune-associated processes, including myeloid cell activation involved in immune response (OR = 3.58, FDR = 0.019) and integrin-mediated signaling pathway (OR = 2.96, FDR = 0.046) were also represented among enriched gene sets. Down-regulated validation gene sets again centered on neuronal and synaptic biology, including synaptic signaling (OR = 2.56, FDR = 1.21 × 10^−8^), vesicle-mediated transport in synapse (OR = 3.91, FDR = 1.13 × 10^−7^), regulation of trans-synaptic signaling (OR = 2.82, FDR = 2.33 × 10^−7^), cell–cell signaling (OR = 2.04, FDR = 4.89 × 10^−7^), synaptic vesicle maturation (OR = 13.10, FDR = 6.36 × 10^−5^), and neurotransmitter secretion (OR = 3.51, FDR < 0.001). These results are consistent with gene set-level suppression of synaptic processes across cohorts, with broader but still overlapping immune-associated upregulation.

GSEA of the validation cohort also showed strong positive enrichment and more limited negative enrichment. A total of 1524 gene sets were positively enriched and 111 were negatively enriched at nominal significance (*p* < 0.05), with 823 positive and 2 negative gene sets remaining significant after FDR correction (Table S7). The most strongly positively enriched gene sets were miRNA transcription (NES = 2.61, FDR < 0.001), negative regulation of the miRNA metabolic process (NES = 2.57, FDR < 0.001), positive regulation of alpha-beta T cell differentiation (NES = 2.56, FDR < 0.001), regulation of the miRNA metabolic process (NES = 2.55, FDR < 0.001), and regulation of small GTPase-mediated signal transduction (NES = 2.51, FDR < 0.001). Lipopolysaccharide-mediated signaling was also strongly positively enriched (NES = 2.49, FDR = 0.002), consistent with persistent immune activation. Negatively enriched gene sets were fewer and weaker in magnitude than in discovery. The strongest included amino acid activation (NES = −1.97, FDR = 0.023) and synaptic vesicle lumen acidification (NES = −1.93, FDR = 0.025), while synaptic vesicle maturation (NES = −1.79, FDR = 0.191), vesicle-mediated transport in synapse (NES = −1.697, FDR = 0.496), and presynaptic endocytosis (NES = −1.62, FDR = 0.865) were nominally but not FDR significant. Overall, the GSEA results in validation cohort support continued enrichment of immune-related processes and more modest negative enrichment of selected synaptic function gene sets.

**Figure 2:**
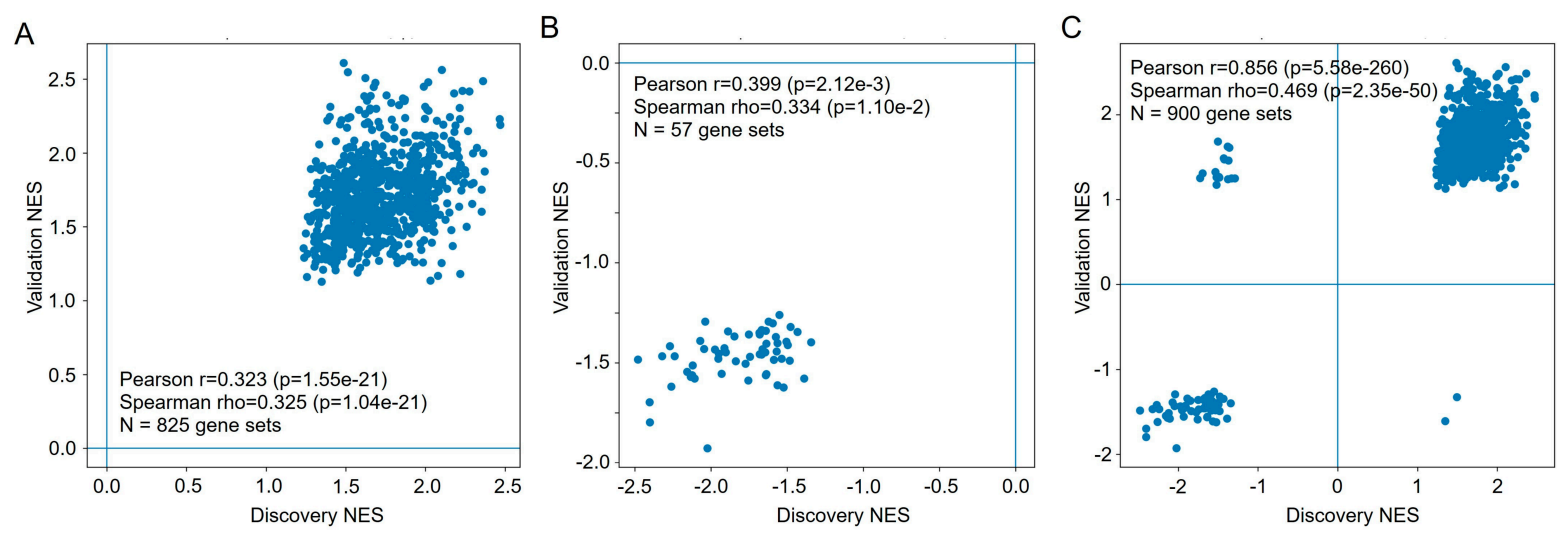
Directional concordance of GSEA normalized enrichment scores (NES) between discovery and validation cohorts. Scatter plots compare GSEA NES for Gene Ontology Biological Process gene sets shared between the discovery dataset (*x*-axis) and the validation dataset (*y*-axis). Horizontal and vertical reference lines indicate NES = 0. (**A**) Shared positively enriched gene sets (N = 825), showing modest but significant concordance in enrichment magnitude between cohorts. (**B**) Shared negatively enriched gene sets (N = 57), also showing significant concordance. (**C**) All shared nominally significant gene sets (N = 900), shown as a pooled descriptive comparison. The pooled correlation is influenced by the separation of positively and negatively enriched gene sets and was therefore not used as the primary measure of concordance strength. Gene sets in the upper-right quadrant represent concordant positive enrichment across cohorts, whereas those in the lower-left quadrant represent concordant negative enrichment. Each point represents a gene set. Pearson correlation (r) and Spearman rank (ρ) correlation coefficients, associated *p*-values, and the number of gene sets (N) are shown.

To quantify gene set-level reproducibility across the two cohorts, NES from GSEA were compared between discovery and validation cohorts for nominally significant shared GSEA gene sets using both Pearson correlation and Spearman rank correlation (Figure 2). Among shared positively enriched gene sets, 825 gene sets overlapped and showed modest but significant concordance in enrichment magnitude (Pearson r = 0.323, *p* = 1.55 × 10^−21^; Spearman ρ = 0.325, *p* = 1.04 × 10^−21^, Figure 2A). Among shared negatively enriched gene sets, 57 gene sets overlapped and also showed significant concordance (Pearson r = 0.399, *p* = 2.12 × 10^−3^; Spearman ρ = 0.334, *p* = 1.10 × 10^−2^, Figure 2B). When all shared nominally significant gene sets were considered together, the pooled correlation was higher across 900 gene sets overlapped (Pearson r = 0.856, *p* = 5.58 × 10^−260^; Spearman ρ = 0.469, *p* = 2.35 × 10^−50^, Figure 2C). However, this pooled value was interpreted descriptively because it is influenced by the separation of positively and negatively enriched gene sets. Therefore, concordance was interpreted primarily from the direction-stratified positive-only and negative-only comparisons. These analyses indicate that although the leading gene sets differ somewhat between cohorts, the broader biological patterns are reproducible. Immune-associated processes were consistently represented among positively enriched gene sets, while neuronal and synaptic processes were consistently represented among negatively enriched gene sets in sCJD brain tissues.

### 2.2. Modest miRNA Expression Changes Exhibit Consistent Downregulation Across Cohorts

To determine whether small non-coding regulatory transcripts exhibit reproducible alterations in sCJD, differential expression analysis of miRNA profiles was performed in internal discovery and validation cohorts derived from GSE140069. As in the mRNA analysis, nominal (*p* < 0.05) and FDR-adjusted thresholds were applied to assess statistical significance, and cross-cohort reproducibility was evaluated using overlap analysis. Given the typically lower dynamic range and smaller effect sizes observed for miRNAs compared to mRNAs, emphasis was placed on directionality and consistency across datasets rather than absolute DEG counts.

#### 2.2.1. Limited Numbers of Differentially Expressed miRNAs Are Reproducible Across Cohorts

**Figure 3:**
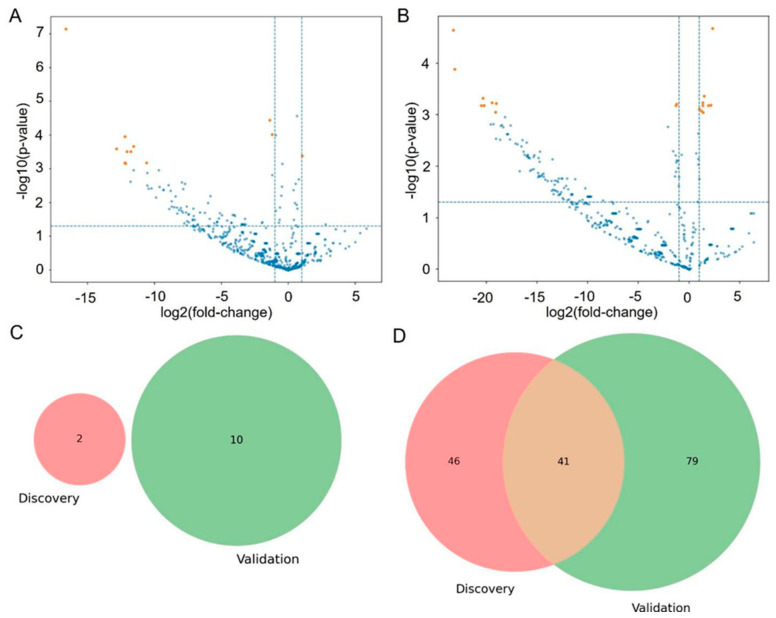
Reproducibility of differential expression across discovery and internal validation miRNA cohorts. (**A**,**B**) Volcano plots of differential miRNA expression in the discovery and internal validation cohorts, respectively. The *x*-axis represents log2(fold change) and the *y*-axis represents -log10(*p*-value). (**C**,**D**) Venn diagrams showing overlap of nominally significant miRNAs between discovery and validation datasets. Panel (**C**) shows over-expressed miRNAs, with no shared transcripts across cohorts. Panel (**D**) shows under-expressed miRNAs, including 41 shared transcripts. In panels (**A**,**B**), vertical dashed lines indicate the fold-change cutoff (|log2FC| > 1), the horizontal dashed line indicates the nominal significance threshold (*p* = 0.05), and orange points indicate miRNAs meeting the fold-change threshold and FDR < 0.05. In panels (**C**,**D**), red circles represent the discovery cohort, green circles represent the validation cohort, and overlapping regions indicate shared miRNAs between cohorts.

Differential expression analysis of the discovery cohort revealed 89 miRNAs that met nominal significance at *p* < 0.05 with |log2FC| > 1 (Figure 3A; Table S8). Of these, 87 were under-expressed and 2 were over-expressed, indicating a marked directional bias toward reduced miRNA expression in sCJD. After FDR-BH correction, 12 miRNAs (11 under-expressed and 1 over-expressed) remained significant at FDR < 0.05.

In the internal validation cohort, 130 miRNAs met the nominal significance threshold (Figure 3B; Table S9), including 120 under-expressed and 10 over-expressed transcripts. Thus, as in the discovery dataset, under-expressed miRNAs substantially outnumbered over-expressed miRNAs. Following multiple testing correction, 19 miRNAs remained significant at FDR < 0.05, of which 10 were under-expressed and 9 were over-expressed transcripts.

Cross-cohort overlap analysis was performed at the miRNA level using nominal *p* < 0.05 thresholds and required concordant directionality. For over-expression, 2 miRNAs were revealed in the discovery cohort and 10 in the internal validation cohort, with none shared between the two datasets (Figure 3C). In contrast, for under-expression, 41 miRNAs were shared between cohorts, while 46 were unique to the discovery and 79 were unique to the internal validation cohorts (Figure 3D, Table 2). Together, these findings indicate that miRNA dysregulation in sCJD is modest in scale relative to the mRNA layer but shows a reproducible directional bias toward downregulation across the two cohorts.

To assess whether these miRNA signatures captured sCJD-related patterns in the internal validation dataset, PCA was performed in the validation cohort using miRNA subsets defined from the discovery dataset at nominal significance (Figure S4). The over-expressed subset showed little separation between sCJD and control samples, despite the first two principal components accounting for all observed variance in this small feature set (PC1 = 55.2%, PC2 = 44.8%). In contrast, the under-expressed miRNA subset showed clearer, although still incomplete, group separation, with the first two components explaining 40.9% of total variance (PC1 = 33.8%, PC2 = 7.10%). Since the over-expressed subset contained few miRNAs, the high proportion of variance explained by PC1 and PC2 should be interpreted cautiously and does not by itself indicate a robust validation pattern.

Classification performance was then evaluated in the validation cohort using logistic regression with LOOCV (Figure S5). Models based on under-expressed miRNAs performed better than those based on over-expressed miRNAs, with AUC ROC values of 0.717 and 0.389, respectively. Random forest models with repeated stratified k-fold validation showed a similar pattern (Figure S6), with AUC ROC values of 0.772 for under-expressed miRNAs and 0.539 for over-expressed miRNAs. Complementary descriptive classifier metrics are provided in Table S3, including sensitivity, specificity, precision, F1-score, PR-AUC, and AUC ROC. Collectively, these analyses indicate that reproducible miRNA alterations in sCJD are dominated by under-expressed transcripts and provide modest discriminatory power, although this signal remains substantially weaker than that observed in the mRNA transcriptomic layer.

#### 2.2.2. Functional Enrichment of Predicted miRNA Targets Shows Limited Cross-Cohort Reproducibility

To determine whether differential miRNA expression was associated with biological processes, functional enrichment analyses were performed on predicted mRNA targets of differentially expressed miRNAs in both discovery and validation cohorts. As in the mRNA analysis, both FET and GSEA were conducted using GO-BP gene sets. Nominal *p* < 0.05 thresholds were used for gene set overlap and cross-cohort comparison analyses to maintain consistency with the broader integrative framework.

In the discovery cohort, FET revealed 183 GO-BP gene sets, of which 76 were down-regulated and 107 were up-regulated at nominal significance (Table S10). None of the down-regulated gene sets survived multiple-testing correction, whereas 45 up-regulated gene sets remained significant at FDR < 0.05. The down-regulated gene sets were heterogeneous and represented transcription initiation (OR = 14.18, FDR = 0.385), wound-response regulation (OR = 31.47, FDR = 0.385), sensory organ development (OR = 3.67, FDR = 0.385), neural tube development (OR = 8.48, FDR = 0.385), and broader morphogenetic processes such as cranial nerve morphogenesis (OR = 10.30, FDR = 0.385), indicating no single dominant biological theme. In contrast, the up-regulated gene sets were driven largely by small overlaps, frequently involving single genes such as ABCC8, and represented cell junction disassembly (OR = complete overlap, FDR = 0.036), glutamate secretion (OR = complete overlap, FDR = 0.036), neuroblast migration (OR = complete overlap, FDR = 0.036), and tight junction organization (OR = complete overlap, FDR = 0.036). These findings indicate that miRNA target enrichment in the discovery cohort is asymmetric, with a statistically stronger up-regulated signal but one that is often based on sparse gene overlap.

GSEA of the discovery target-gene lists revealed 35 nominally significant gene sets, including 34 negatively enriched and one positively enriched gene set (Table S11). After multiple-testing correction, no positively enriched gene set and only two negatively enriched gene sets remained significant at FDR < 0.05. The strongest negatively enriched gene sets were anterior–posterior pattern specification (NES = −1.89, FDR = 0.028), regulation of body fluid levels (NES = −1.85, FDR = 0.026), protein maturation (NES = −1.80, FDR = 0.052), head development (NES = −1.74, FDR = 0.105), and the pattern specification process (NES = −1.68, FDR = 0.125). These negatively enriched gene sets were dominated by developmental and morphogenetic processes rather than immune-regulatory gene sets as observed in mRNA. The only positively enriched gene set was defense response to bacterium (NES = 1.61, FDR = 0.315). Thus, in the discovery dataset, GSEA results are consistent with a modest pattern of negative enrichment centered on developmental processes, with limited evidence for positive enrichment.

In the validation cohort, FET revealed 24 down-regulated and 265 up-regulated GO-BP gene sets at nominal significance (Table S12). As in discovery, none of the down-regulated gene sets survived FDR correction, whereas 17 up-regulated gene sets remained significant. The top up-regulated gene sets in the validation cohort were strongly enriched for developmental and morphogenetic processes, including anterior–posterior pattern specification (OR = 55.17, FDR = 0.007), segmentation (OR = 57.17, FDR = 0.017), anatomical structure formation involved in morphogenesis (OR = 20.8, FDR = 0.017), the pattern specification process (OR = 27.08, FDR = 0.017), embryonic morphogenesis (OR = 24.92, FDR = 0.017), head development (OR = 24.92, FDR = 0.017), and forebrain development (OR = 28.28, FDR = 0.036). In contrast, the down-regulated gene sets in the validation cohort were led by snRNA metabolic and processing gene sets (OR = complete overlap, FDR = 0.438), NADH dehydrogenase complex assembly (OR = complete overlap, FDR = 0.438), transcription initiation (OR = 10.99, FDR = 0.673), and selected metabolic processes such as the long-chain fatty acid biosynthetic process (OR = 8.13, FDR = 0.673). Exact overlap between cohorts was limited, although 14 down-regulated and 8 up-regulated GO-BP gene sets were shared at the nominal level. Overall, the validation FET results indicate partial replication and are more strongly associated with developmental-pattern enrichment than with reproducible immune-regulatory signatures as observed in mRNA cohorts.

GSEA in the validation cohort likewise showed limited reproducibility (Table S13). A total of 12 up-regulated and 16 down-regulated gene sets were nominally significant, but none remained significant after FDR correction. The top positively enriched gene sets included cell fate commitment (NES = 1.96, FDR = 0.088), muscle contraction (NES = 1.79, FDR = 0.150), anterior–posterior pattern specification (NES = 1.62, FDR = 0.350), renal system development (NES = 1.55, FDR = 0.413), and sensory organ development (NES = 1.51, FDR = 0.289). The top negatively enriched gene sets included regulation of proteolysis (NES = −1.60, FDR = 0.177), establishment of protein localization (NES = −1.50, FDR = 0.625), detection of stimulus (NES = −1.46, FDR = 0.718), ribonucleoprotein complex biogenesis (NES = −1.44, FDR = 0.733), and protein transport (NES = −1.40, FDR = 0.741). These results indicate nominal gene set-level shifts in validation, but the dominant themes differ from those observed in discovery and do not provide consistent evidence for shared immune-regulatory processes.

Cross-cohort reproducibility of GSEA gene sets directionality was minimal (Figure S7). Only one down-regulated gene set overlapped between discovery and validation, precluding meaningful correlation analysis for the negative subset. Across all overlapping nominally significant gene sets, only six gene sets were shared, and their NES were not concordant between cohorts (Pearson r = −0.280, *p* = 0.591; Spearman ρ = −0.257, *p* = 0.623). Taken together, these findings indicate that predicted miRNA target enrichment shows limited gene set-level reproducibility across cohorts. While developmental and morphogenetic themes recur in parts of the analysis, the overall signal is weaker and less consistent than that observed in the transcriptomic layer.

### 2.3. DNA Methylation Analysis Reveals Widespread but Weakly Reproducible Epigenetic Changes

To evaluate whether epigenetic alterations are reproducibly associated with sCJD, genome-wide DNA methylation profiles from GSE156994 were analyzed using a discovery–validation framework analogous to the transcriptomic and miRNA analyses. The dataset was randomly divided into discovery and validation cohorts, comprising 75% and 25% of samples, respectively. Differential methylation signatures were first identified in the discovery cohort and subsequently evaluated in the internal validation cohort. Analyses were conducted at the individual CpG-site level using both FDR-adjusted and nominal *p*-value thresholds, and cross-cohort reproducibility was assessed using directional overlap and effect-size concordance approaches. Given the high dimensionality of methylation arrays and typically modest Δβ effect sizes, emphasis was placed on consistency of direction and reproducibility across subsets rather than absolute CpG counts alone.

#### 2.3.1. Numerous CpG Sites Exhibit Differential Methylation with Limited Validation Overlap

**Figure 4:**
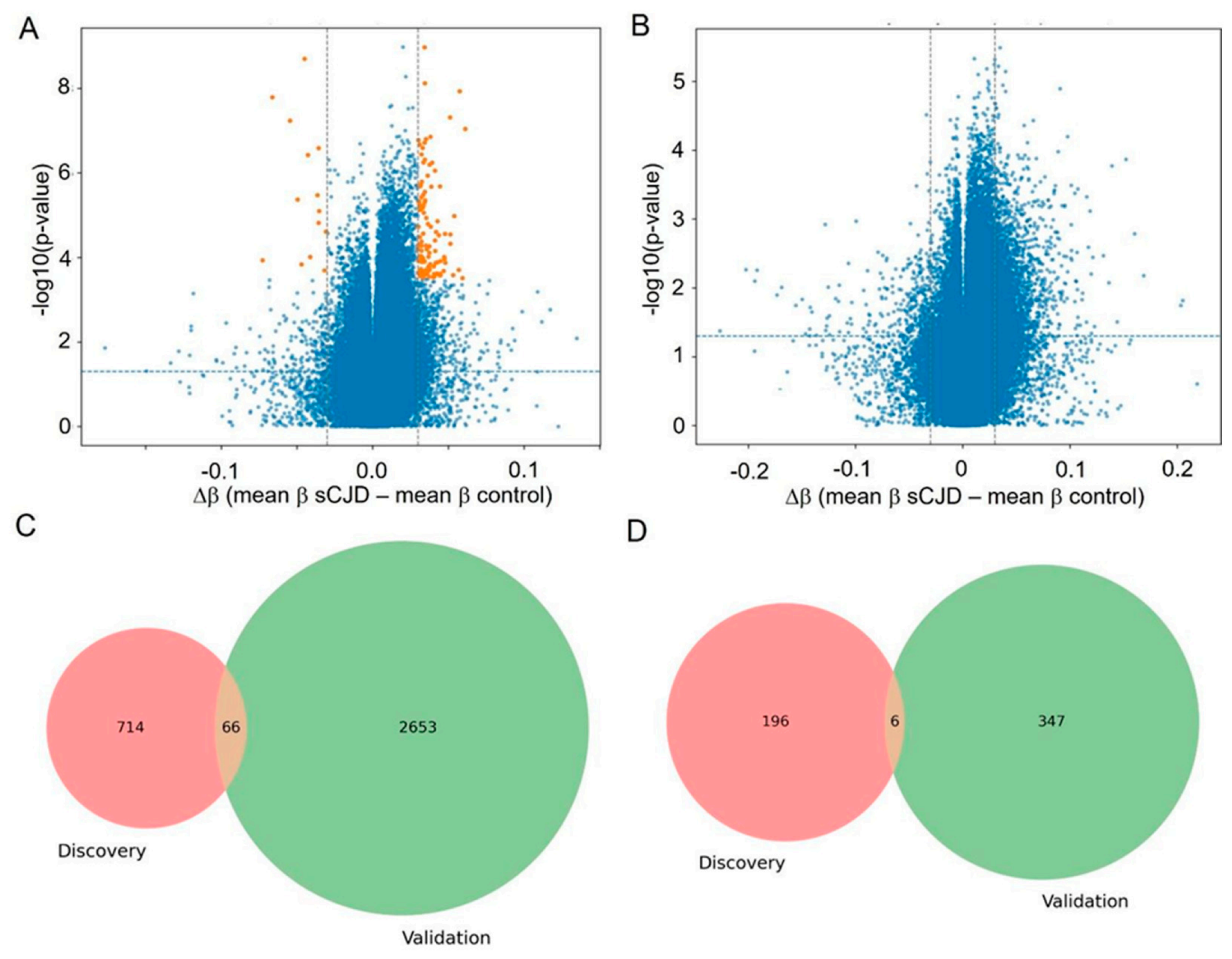
Differential DNA methylation and cross-cohort overlap of CpG sites in sCJD. (**A**) Volcano plot of differentially methylated CpG sites in the discovery cohort (GSE156994). Each point represents a CpG site plotted by Δβ (mean β-sCJD − mean β-control) versus –log10(*p*-value). (**B**) Volcano plot of differentially methylated CpG sites in the internal validation cohort using the same thresholds as discovery. (**C**) Venn diagram showing overlap of nominally significant (*p* < 0.05) hyper-methylated CpG sites between discovery and internal validation datasets. (**D**) Venn diagram showing overlap of nominally significant (*p* < 0.05) hypo-methylated CpG sites between cohorts. Nominal thresholds were used for overlap analyses to maintain consistency with downstream concordance assessments. In panels (**A**,**B**), the horizontal dashed lines indicate the nominal significance (*p* = 0.05), and vertical dashed lines indicate the Δβ thresholds at −0.03 and +0.03, and orange points indicate CpG sites with FDR < 0.05. In panels (**C**,**D**), red circles represent the discovery cohort, green circles represent the validation cohort, and overlapping regions indicate shared CpG sites between cohorts.

Differential methylation analysis of the discovery cohort of GSE156994 revealed widespread CpG-level alterations between sCJD cases and controls. At a nominal significance threshold (*p* < 0.05), 982 CpG sites were differentially methylated, comprising 780 hyper-methylated (positive Δβ) and 202 hypo-methylated (negative Δβ) sites (Table S14). After multiple-testing correction using FDR-BH, 123 CpG sites (109 hyper- and 14 hypo-methylated) remained significant at FDR < 0.05. These results indicate widespread but modest epigenetic differences between sCJD and control, with a pronounced directional skew toward increased methylation in sCJD. The volcano plot of the discovery cohort illustrates the distribution of CpG-level methylation differences across the methylome (Figure 4A). Most CpG sites clustered near Δβ ≈ 0, reflecting small absolute methylation changes typical of complex neurodegenerative disorders, while a subset of loci exhibited stronger statistical support and larger effect sizes. Both hyper-methylated and hypo-methylated CpGs were observed, indicating bidirectional epigenetic dysregulation.

In the internal validation cohort, 3072 CpG sites were differentially methylated, including 2719 hyper-methylated and 353 hypo-methylated sites at the nominal level (*p* < 0.05), though no CpG sites remained significant following FDR correction (Table S15). The volcano plot of the validation cohort demonstrated a similar overall Δβ distribution, suggesting broadly comparable methylation variance signatures between cohorts despite reduced statistical significance after correction (Figure 4B).

To assess reproducibility, overlap analysis was performed using nominally significant CpG sites (*p* < 0.05) and requiring concordant directionality of methylation change. Venn diagram analysis demonstrated limited cross-cohort overlap for both hyper-methylated and hypo-methylated CpGs (Figure 4C,D). Among hyper-methylated CpGs, 66 sites were shared between discovery and validation cohorts, whereas only 6 hypo-methylated CpGs overlapped (Table 3). The majority of nominally significant CpGs were unique to one cohort, indicating limited reproducibility at the individual CpG level. These findings suggest that although widespread methylation differences are detectable in sCJD, many loci show small effect sizes and substantial variability across datasets.

To assess whether aggregate methylation patterns capture sCJD-associated signatures, PCA was performed using CpG subsets revealed in the discovery dataset (Figure S8). PCA based on hyper-methylated CpGs showed modest separation between sCJD and control samples, whereas hypo-methylated subsets exhibited greater overlap. The first two components together explained the majority of variance (hyper-methylated CpGs: PC1 = 9.10%, PC2 = 5.10%; hypo-methylated CpGs: PC1 = 6.00%, PC2 = 5.20%). Classification performance was further evaluated using logistic regression with LOOCV (Figure S9). Models based on nominally significant hypo-methylated CpGs achieved the strongest performance (AUC ROC = 0.774), whereas hyper-methylated CpGs showed near-chance discrimination (AUC ROC = 0.538). Random forest classifiers produced broadly similar patterns (Figure S10). Models based on nominally significant hypo-methylated CpGs yielded the highest discrimination (AUC ROC = 0.875), while hyper-methylated CpGs produced weaker performance (AUC ROC = 0.676). Additional descriptive classifier metrics are summarized in Table S3, including sensitivity, specificity, precision, F1-score, PR-AUC, and AUC ROC. Overall, these analyses indicate that although individual CpG-level reproducibility is limited, aggregate methylation patterns retain modest discriminatory capacity, consistent with smaller Δβ effect sizes and greater variability in high-dimensional epigenetic data.

#### 2.3.2. Methylation-Associated Gene Sets Highlight Cell Adhesion, Neurodevelopment, and Translational Processes

To determine whether CpG-level methylation changes are associated with biological processes, functional enrichment analyses were performed using both FET and GSEA, consistent with the analytic framework applied to the transcriptomic and miRNA layers. Again, the same GO-BP gene sets and statistical thresholds were used to maintain cross-omic comparability.

In the discovery cohort, FET revealed 342 nominally enriched GO-BP gene sets at *p* < 0.05, including 210 associated with hyper-methylated CpGs and 132 associated with hypo-methylated CpGs (Table S16). Of these, 4 gene sets associated with hyper-methylated CpGs and 17 associated with hypo-methylated CpGs remained significant after Benjamini–Hochberg correction at FDR < 0.05. The most strongly enriched gene sets were dominated by adhesion-related biological processes, including homophilic cell adhesion via plasma membrane adhesion molecules (OR = 14.47, FDR = 1.44 × 10^−21^), cell–cell adhesion via plasma membrane adhesion molecules (OR = 8.67, FDR = 2.92 × 10^−17^), cell adhesion (OR = 3.04, FDR = 9.25 × 10^−11^), and cell–cell adhesion (OR = 3.42, FDR = 5.43 × 10^−10^). Additional nominally enriched GO-BP terms represented neurobiology and signaling processes, including synaptic signaling (OR = 1.95, FDR = 0.341), radial glia-guided pyramidal neuron migration (OR = 45.89, FDR = 0.106), and selected antigen-presentation processes such as exogenous antigen presentation via MHC class II (OR = 7.99, FDR = 0.591). These findings indicate that methylation-associated gene sets in the discovery dataset were primarily related to cell adhesion and neurobiologically relevant regulation, rather than to a diffuse set of unrelated processes.

GSEA of the discovery cohort revealed 582 nominally enriched GO-BP gene sets, including 440 positively enriched and 142 negatively enriched gene sets (Table S17). Two positively enriched and four negatively enriched gene sets remained significant after FDR correction. The strongest positively enriched gene sets again emphasized adhesion and neuronal signaling processes, including homophilic cell adhesion via plasma membrane adhesion molecules (NES = 2.71, FDR = 0.002), cell–cell adhesion via plasma membrane adhesion molecules (NES = 2.37, FDR = 0.046), nucleus localization (NES = 2.21, FDR = 0.153), response to L-glutamate (NES = 2.15, FDR = 0.180), and postsynaptic density assembly (NES = 2.13, FDR = 0.186). In contrast, negatively enriched gene sets were dominated by ribosome and RNA-processing processes, including ribosomal large subunit biogenesis (NES = −2.87, FDR < 0.001), ribosome biogenesis (NES = −2.76, FDR = 0.002), ribonucleoprotein complex biogenesis (NES = −2.46, FDR = 0.022), and cytoplasmic translation (NES = −2.30, FDR = 0.051), together with the sensory-perception gene sets such as smell (NES = −2.91, FDR < 0.001). These results are consistent with methylation-associated patterns involving suppression of adhesion and neuronal signaling processes alongside activation of ribosomal and translational processes in the discovery dataset.

In the internal validation cohort, FET revealed 816 nominally enriched GO-BP gene sets, comprising 658 associated with hyper-methylated CpGs and 158 associated with hypo-methylated CpGs (Table S18). Among these, 111 gene sets associated with hyper-methylated CpGs and 3 associated with hypo-methylated CpGs remained significant after FDR correction. The strongest enriched gene sets again centered on adhesion-related biological processes, including homophilic cell adhesion via plasma membrane adhesion molecules (OR = 6.54, FDR = 5.61 × 10^−17^), cell–cell adhesion via plasma membrane adhesion molecules (OR = 4.38, FDR = 1.09 × 10^−14^), cell adhesion (OR = 2.12, FDR = 4.85 × 10^−13^), and cell–cell adhesion (OR = 2.44, FDR = 1.96 × 10^−13^). Validation enrichment also highlighted neurodevelopmental and morphogenetic processes such as neurogenesis (OR = 1.87, FDR = 2.35 × 10^−9^), generation of neurons (OR = 1.93, FDR = 2.74 × 10^−9^), embryonic morphogenesis (OR = 2.30, FDR = 1.35 × 10^−7^), sensory organ development (OR = 2.26, FDR = 3.04 × 10^−7^), and pattern specification processes (OR = 2.38, FDR = 3.25 × 10^−6^). Thus, although individual CpG overlap between cohorts was limited, gene set-level enrichment patterns in the validation dataset showed similar adhesion and neurodevelopmental themes observed in discovery.

GSEA of the internal validation cohort revealed 1112 nominally enriched GO-BP gene sets, including 771 positively enriched and 341 negatively enriched gene sets (Table S19). Forty-nine positively enriched and 120 negatively enriched gene sets remained significant after FDR correction. The strongest positively enriched gene sets again emphasized adhesion and neuronal-recognition processes, including homophilic cell adhesion via plasma membrane adhesion molecules (NES = 3.41, FDR < 0.001), regulation of cell–substrate junction organization (NES = 2.60, FDR < 0.001), cell–cell adhesion via plasma membrane adhesion molecules (NES = 2.59, FDR < 0.001), regulation of cell–matrix adhesion (NES = 2.45, FDR = 0.004), and neuron recognition (NES = 2.43, FDR = 0.004). Negatively enriched gene sets were dominated by ribosomal, RNA-processing, and mitochondrial translational processes, including ribosome biogenesis (NES = −2.83, FDR = 0.003), ribosome assembly (NES = −2.83, FDR < 0.001), mitochondrial translation (NES = −2.82, FDR < 0.001), rRNA metabolic processes (NES = −2.75, FDR < 0.001), rRNA processing (NES = −2.75, FDR < 0.001), and ribonucleoprotein complex biogenesis (NES = −2.67, FDR = 0.003), as well as mitochondrial gene expression (NES = −2.65, FDR = 0.003) and cell-cycle checkpoint signaling (NES = −2.39, FDR = 0.009). These results indicate that the same broad contrast between adhesion-related processes and ribosomal or translational processes is present in both cohorts.

To evaluate reproducibility of methylation-associated gene set enrichment, NES for nominally significant gene sets were compared between discovery and internal validation cohorts (Figure S11). Cross-cohort concordance was statistically significant overall. Among negatively enriched gene sets, 192 overlapping gene sets demonstrated significant Pearson correlation between cohorts (r = 0.343, *p* = 1.10 × 10^−6^), although rank-based correlation was weaker (Spearman ρ = 0.112, *p* = 0.122). Among positively enriched gene sets, 67 overlapping gene sets showed significant concordance by both Pearson (r = 0.408, *p* = 6.09 × 10^−4^) and Spearman rank correlations (ρ = 0.276, *p* = 0.0237). When all overlapping gene sets were considered together, the pooled correlation was higher (Pearson r = 0.853, *p* = 4.11 × 10^−79^; Spearman ρ = 0.550, *p* = 4.01 × 10^−23^). However, this pooled value was interpreted descriptively because it is influenced by the separation of positively and negatively enriched gene sets. Therefore, methylation-associated gene set reproducibility was interpreted primarily from the direction-stratified comparisons. These findings indicate that gene set-level methylation signals show reproducible directional signatures despite limited overlap at the level of individual CpG sites.

### 2.4. Cross-Omic Comparison Shows Limited FDR-Supported Overlap Across Molecular Layers

**Figure 5:**
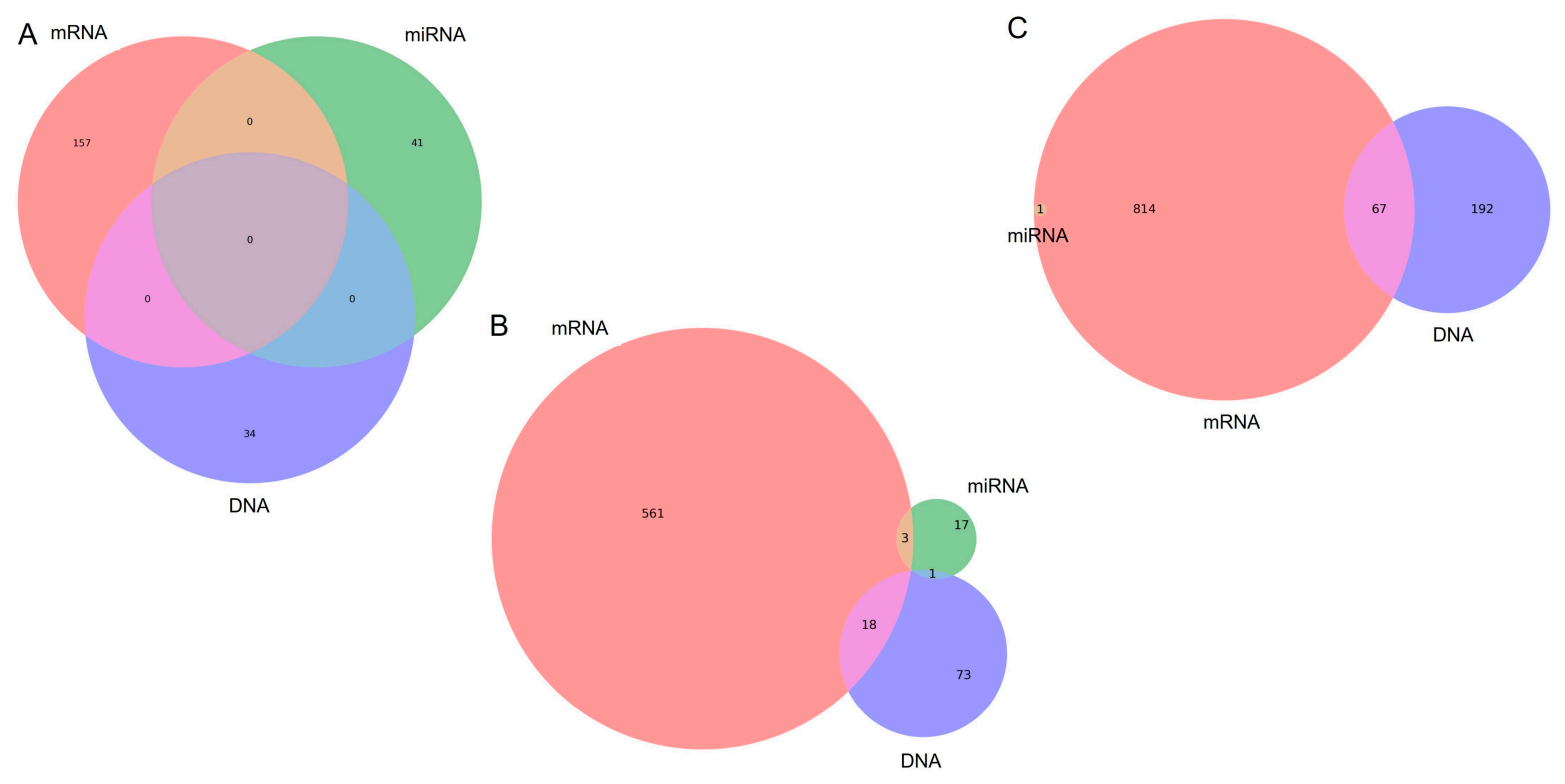
Cross-omic overlap of validated genes and biological gene sets across transcriptomic, miRNA, and DNA methylation layers. Venn diagrams summarize the overlap of features revealed at nominal significance (*p* < 0.05) in both discovery and validation cohorts within each omic layer. In all Venn diagrams, red represents mRNA, green represents miRNA, and blue represents DNA methylation; overlapping regions represent features or gene sets shared between the corresponding molecular layers. (**A**) Gene-level overlap. Genes were unique to individual layers, and no genes were shared between layers, indicating minimal cross-omic convergence at the level of individual gene features. (**B**) Overrepresentation analysis (FET) gene sets. The majority of enriched Gene Ontology Biological Process gene sets were layer-specific, although modest overlap was observed between mRNA and DNA methylation datasets and between mRNA and miRNA datasets, with a single gene set shared between miRNA and DNA layers. No gene sets were shared across all three omic layers. (**C**) Gene set enrichment analysis (GSEA) gene sets. Most gene sets were again unique to individual layers, with 67 gene sets shared between mRNA and DNA methylation datasets and no overlap involving miRNA-derived gene sets. Together, these exploratory nominal-threshold analyses suggest that any cross-omic gene set-level recurrence is most apparent between transcriptomic and DNA methylation datasets.

To evaluate whether molecular alterations in sCJD are shared across transcriptomic, miRNA, and DNA methylation layers, cross-omic comparison was performed at three hierarchical levels: individual genes, gene sets identified by overrepresentation testing (FET), and gene sets identified by GSEA. Union sets of shared genes and gene sets were constructed separately for RNA, miRNA, and DNA methylation datasets and compared using set intersections. Overlap was summarized descriptively and visualized using Venn diagrams (Figure 5).

Since threshold choice can strongly affect overlap-based comparisons, cross-omic gene set overlap was evaluated using both FDR-adjusted and nominal *p*-value thresholds. FDR-adjusted overlap was treated as the primary analysis, while nominal *p* < 0.05 overlap was considered exploratory (Table 4). Under the FDR-adjusted threshold, no cross-omic gene set overlap was observed between mRNA, miRNA, and DNA methylation layers for either FET or GSEA. Under the exploratory nominal threshold, overlap was observed mainly between mRNA and DNA methylation datasets, with 18 shared FET gene sets and 67 shared GSEA gene sets. Smaller or no overlaps were observed for comparisons involving the miRNA layer.

At the gene level, reproducible signals were largely specific to individual molecular layers (Figure 5A). Among shared genes revealed within each molecular layer, most were layer-specific. mRNA contributed the largest set, with 157 genes uniquely revealed in the transcriptomic dataset. miRNA target analysis yielded 41 unique genes, and DNA methylation analysis revealed 34 unique genes. No genes were shared between mRNA and miRNA, mRNA and DNA methylation, or miRNA and DNA methylation, and no genes were common across all three layers. This absence of gene-level overlap should be interpreted cautiously. The datasets differed in tissue source and experimental platform, with mRNA data derived from the frontal cortex and miRNA and DNA methylation data derived from whole blood. In addition, each molecular layer measured different feature types and required different annotation steps before comparison at the gene level. Therefore, the lack of shared genes likely reflects, at least in part, cross-tissue and cross-platform differences in detectable feature space, rather than demonstrating that shared gene-level biology is absent in sCJD.

Using the exploratory nominal threshold, FET-based gene set-level analyses revealed cross-layer overlap across molecular layers, particularly between transcriptomic and DNA methylation datasets. Most enriched GO-BP gene sets remained layer-specific (Figure 5B). mRNA contained 561 unique gene sets, DNA methylation contained 73 unique gene sets, and miRNA contained 17 unique gene sets. However, 18 gene sets overlapped between mRNA and DNA methylation datasets, and 3 gene sets overlapped between mRNA and miRNA datasets (Table 5). Only a single gene set was shared between miRNA and DNA methylation layers, and no gene sets were common to all three layers.

GSEA produced a similar exploratory nominal-threshold pattern, with the strongest overlap observed between mRNA and DNA methylation datasets (67 gene sets; Figure 5C). The majority of gene sets were unique to individual layers, with 814 gene sets specific to mRNA, 192 gene sets specific to DNA methylation, and one gene set unique to miRNA. Sixty-seven gene sets overlapped between mRNA and DNA methylation datasets, whereas no gene sets were shared between mRNA and miRNA or between miRNA and DNA methylation layers.

Taken together, FDR-supported cross-omic gene set overlap was not observed in the present analysis. Exploratory nominal-threshold overlap was detected mainly between transcriptomic and DNA methylation datasets, while comparisons involving the miRNA layer showed little or no overlap. These findings provide the basis for the cross-method gene set comparison described in the following section.

### 2.5. Cross-Method Comparison Highlights Exploratory Adhesion-Related Gene Set Recurrence

To determine whether exploratory nominal-threshold gene set signals were supported by more than one enrichment framework, gene sets identified by both FET and GSEA were compared across mRNA, miRNA, and DNA methylation datasets. Gene sets were included if they were nominally significant (*p* < 0.05) in both discovery and validation cohorts within a given molecular layer. This analysis was designed to identify biological processes supported by complementary enrichment approaches, rather than to establish FDR-supported cross-omic convergence.

Across all modalities, 270 unique GO-BP gene sets were supported by both FET and GSEA (Figure S12). The majority of gene sets originated from the mRNA dataset, with 254 unique to mRNA, compared with 14 unique to DNA methylation and one unique to miRNA. One gene set, CELL_SUBSTRATE_ADHESION, appeared in both mRNA and DNA methylation, and no gene sets were shared between miRNA and the other modalities. This pattern indicates that cross-method recurrence was driven mainly by the transcriptomic layer, with limited but notable exploratory overlap between transcriptomic and DNA methylation datasets (Figure S12, Table S20).

The shared mRNA–DNA methylation gene set, GOBP_CELL_SUBSTRATE_ADHESION, was supported by both enrichment frameworks, indicating recurrence of a cell–substrate adhesion signal across transcriptomic and methylation-associated analyses. Because expression and methylation profiles represent distinct regulatory layers, direction labels were treated as layer-specific annotations rather than direct evidence of concordant regulation. Since this signal was identified under nominal-threshold criteria, it should be interpreted as exploratory rather than as definitive evidence of FDR-supported cross-omic convergence. Nevertheless, its detection by both FET and GSEA suggests that adhesion-related biological processes may represent a useful candidate signal for follow-up in future studies.

To examine whether recurrent gene sets formed broader biological processes, we constructed a gene set similarity network focused on the most recurrent terms across molecular layers and enrichment methods. Since the complete set contained hundreds of gene sets, visualization focused on the 20 most recurrent gene sets, defined as those appearing in the greatest number of modality–direction–method combinations.

**Figure 6:**
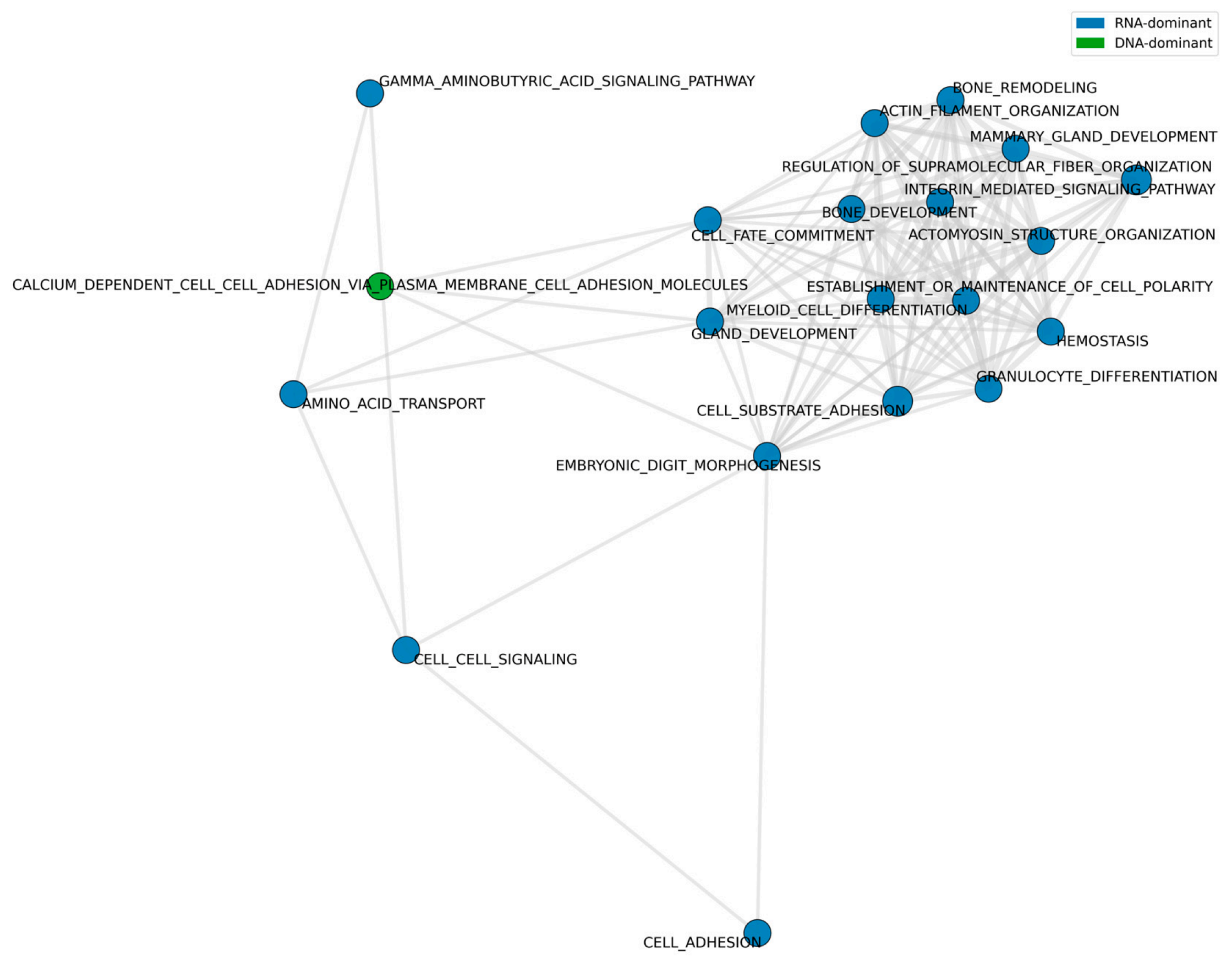
Cross-omic gene set similarity network derived from shared FET and GSEA enrichment signals. Network representation of the top recurrent Gene Ontology Biological Process (GO-BP) gene sets identified across transcriptomic, miRNA, and DNA methylation analyses. Nodes represent gene sets reproduced by both overrepresentation analysis (FET) and preranked gene set enrichment analysis (GSEA) within at least one molecular layer. Edges represent Jaccard similarity between gene sets, indicating shared functional context. Node color indicates the omic layer in which the gene set signal was most prominent (RNA, blue; DNA methylation, green). The network reveals a dense cluster of RNA-dominant gene sets related primarily to cell adhesion, cytoskeletal organization, and developmental regulation, with smaller peripheral components associated with DNA methylation signals. The limited number of gene sets reproduced by both enrichment frameworks illustrates low methodological redundancy between FET and GSEA, while the clustering of related processes indicates higher-order biological convergence across analytical perspectives.

The resulting network showed clustering of biologically related processes rather than uniform connectivity (Figure 6). mRNA-associated gene sets formed the largest connected component, reflecting the dominant contribution of transcriptomic enrichment results. Recurrent processes included cytoskeletal organization and cell adhesion, represented by ACTIN_FILAMENT_ORGANIZATION, ACTOMYOSIN_STRUCTURE_ORGANIZATION, CELL_SUBSTRATE_ADHESION, and CELL_CELL_SIGNALING, as well as developmental and differentiation processes including EMBRYONIC_DIGIT_MORPHOGENESIS, GLAND_DEVELOPMENT, BONE_DEVELOPMENT, and GRANULOCYTE_DIFFERENTIATION. Additional recurrent gene sets represented tissue remodeling, structural regulation, signaling, and metabolic processes, including BONE_REMODELING, MAMMARY_GLAND_DEVELOPMENT, and REGULATION_OF_SUPRAMOLECULAR_FIBER_ORGANIZATION, GAMMA_AMINOBUTYRIC_ACID_SIGNALING, and AMINO_ACID_TRANSPORT.

Together, these findings indicate that although exact gene set identities showed limited overlap across molecular layers, cross-method comparison identified related biological themes supported by both FET and GSEA. The most consistent signal arose from the transcriptomic layer, while the limited mRNA–DNA methylation overlap highlighted cell–substrate adhesion as an exploratory gene set-level signal. These results support the use of cross-method gene set comparison as a hypothesis-generating approach for identifying biologically coherent processes in heterogeneous public sCJD datasets.

## 3. Discussion

sCJD is a rapidly progressive and fatal neurodegenerative disorder characterized by misfolded prion protein accumulation, neuronal loss, spongiform degeneration, and extensive gliosis. Human neuropathological and molecular studies have consistently demonstrated inflammatory activation and synaptic dysfunction across affected brain regions [13,19,20]. In this context, cross-omic comparative analysis provides an opportunity to evaluate whether disease-associated molecular signals recur across independently generated transcriptomic, miRNA, and DNA methylation datasets, either at the level of individual molecular features or at the broader level of biological processes.

The central message of this study is that heterogeneous public sCJD datasets are unlikely to reveal a single conserved gene-level molecular signature across tissues and platforms, but they can still be useful for identifying reproducible bulk-tissue signatures and cautious biological-process hypotheses derived from GO-BP gene set-level analyses. The strongest reproducible signal was the cortical transcriptomic pattern of immune-associated activation and synaptic or neuronal suppression. In contrast, cross-omic adhesion-related recurrence was not supported as a universal FDR-controlled signal across all molecular layers, but appeared in exploratory nominal-threshold analyses, mainly between transcriptomic and DNA methylation datasets. Therefore, the value of this study lies in defining where reproducible molecular signal is strongest, where cross-layer evidence is weak, and which gene set-level hypotheses may justify follow-up in better matched datasets.

The main finding of this study is not strong gene-level convergence across molecular layers, but rather limited feature-level overlap together with cautious gene set-level recurrence. This distinction is important because the analyzed datasets differed in tissue source, platform, cohort structure, and analyzable molecular space. The mRNA datasets were derived from post-mortem frontal cortices, whereas the miRNA and DNA methylation datasets were derived from whole blood. These tissues differ substantially in cellular composition, expressed genes, regulatory activity, and assay-accessible feature space. In addition, transcriptomic, miRNA, and DNA methylation platforms measure different molecular features and require different annotation steps before comparison at the gene level. Therefore, the absence of gene-level overlap across mRNA, miRNA, and DNA methylation layers should not be interpreted as evidence that sCJD lacks shared molecular biology. Rather, it reflects, at least in part, the constraints of comparing heterogeneous public datasets generated from different tissues and technologies.

The most reproducible molecular signals arose from the transcriptomic layer. Differential expression analysis identified genes with consistent directionality across the two transcriptomic cohorts, and GO-BP gene set-level analyses showed substantial concordance between discovery and validation datasets. These findings are consistent with previous human studies of prion disease. Regional transcriptomic profiling of sCJD brains has demonstrated substantial activation of inflammatory and immune-related processes, with subtype-specific differences between MM1 and VV2 disease [21,22]. Transcriptomic analyses have also highlighted disruption of synaptic signaling, vesicle trafficking, and neuronal maintenance [17]. The present study extends this work by showing that these broad transcriptomic patterns are reproducible across independent cohorts, even when the exact genes contributing to the signals differ between datasets.

However, the recurrent immune-up and synaptic-down transcriptomic pattern should be interpreted in the context of bulk post-mortem cortical tissue. sCJD is characterized by severe neuronal loss, spongiform change, and reactive gliosis, and these pathological changes are expected to alter the cellular composition of the sampled cortex. Therefore, immune-associated up-regulation and synaptic or neuronal down-regulation may reflect shifts in cell-type proportions, including increased glial contribution and reduced neuronal signal, rather than purely cell-intrinsic transcriptional regulation. We did not perform cell-type deconvolution because the study was designed as a harmonized cross-dataset comparison of publicly available molecular profiles generated on different platforms, and reference-based deconvolution would introduce an additional layer of assumptions related to reference selection, tissue compatibility, platform coverage, and cell-type marker stability in diseased post-mortem cortices. Accordingly, these transcriptomic results are best interpreted as reproducible bulk-tissue disease signatures rather than definitive evidence of cell-intrinsic immune activation or neuronal transcriptional suppression.

In contrast to the transcriptomic layer, miRNA and DNA methylation datasets showed weaker feature-level reproducibility and limited overlap with transcriptomic signals. Previous studies have reported altered miRNA expression in both brain tissue and biofluids from prion disease patients, although the specific miRNAs implicated vary considerably between studies and biological matrices [23]. Similarly, altered DNA methylation profiles have been reported in peripheral blood from individuals with sCJD, including disease-associated CpG loci with potential diagnostic or prognostic relevance [19]. In the present analysis, miRNA and methylation results provided useful comparative context, but did not support strong cross-layer convergence at the individual feature level. This uneven distribution of reproducible signal across data types is consistent with the challenges of comparing molecular layers generated from different tissues, platforms, and regulatory feature spaces.

For the miRNA layer, the one-to-one miRNA-to-gene mapping strategy was used to reduce redundancy and allow miRNA results to be compared with gene-level transcriptomic and DNA methylation results. This choice made it possible to incorporate the miRNA dataset into the same gene set framework used for the other molecular layers. However, individual miRNAs can regulate many target transcripts, and individual genes can also be regulated by multiple miRNAs. Therefore, assigning each miRNA to a single representative target simplifies the many-to-many structure of miRNA regulation and likely underestimates the broader regulatory footprint of the miRNA layer. For this reason, the limited miRNA overlap observed here should not be interpreted as evidence that miRNA regulation is unimportant in sCJD. Rather, it indicates that the conservative target-mapping strategy used here limits the biological resolution of the miRNA analysis. Future analyses using full target sets, target-weighted enrichment, or dedicated miRNA pathway methods would be better suited to evaluating miRNA-associated regulatory pathways in sCJD.

The DNA methylation analysis was limited to individual CpG-level testing followed by probe-to-gene annotation for gene set analysis. We did not aggregate CpGs into differentially methylated regions or intersect CpG coordinates with regulatory annotations such as promoters, enhancers, CpG islands, or CpG shores. The analysis also did not remove cross-reactive or SNP-overlapping Illumina 450K probes before differential testing. Although the main methylation findings were interpreted at the gene set-level, unfiltered probes may introduce probe-specific noise or artifacts into CpG-level differential methylation results. Therefore, the limited CpG-level overlap observed here should not be interpreted as evidence that reproducible methylation-associated regulatory signals are absent in sCJD. Future analyses using DMR-based, regulatory annotation-based, and probe-filtered approaches may better capture reproducible epigenetic signals. Despite limited feature-level overlap, GO-BP gene set-level analyses suggested broader biological-process recurrence, particularly between transcriptomic and DNA methylation datasets under exploratory nominal-threshold criteria. Under FDR-controlled cross-layer criteria, broad cross-omic gene set overlap was not observed, and therefore the gene set-level findings should not be considered definitive FDR-supported convergence. However, exploratory nominal-threshold analyses identified recurrent adhesion-related processes, including cell–substrate and cell–cell adhesion, mainly between mRNA and DNA methylation datasets. These findings are best interpreted as hypothesis-generating signals that may point to shared cellular-interaction or tissue-organization processes in sCJD, rather than as confirmed mechanistic pathways.

Although adhesion- and morphogenesis-related pathways are not usually framed as central canonical drivers of prion disease, they are biologically plausible in the context of PrP biology. PrP^C is a cell-surface glycoprotein, and prior work showed that it can associate with neural cell adhesion molecule isoforms, directly linking PrP biology with cell-adhesion and cell-surface interaction processes [24]. However, N-CAM deficiency did not prevent scrapie disease, and CD44 was reported to be dispensable for reactive astrocyte activation during prion disease, indicating that adhesion-related findings should not be interpreted as evidence for a single required adhesion molecule or causal pathway [24,25]. Instead, the adhesion-related signatures observed here may reflect broader cell-surface, extracellular, glial, or tissue-interface responses. Additional studies have connected prion biology with cell adhesion-, extracellular matrix-, and cell-surface-associated processes [26,27,28,29]. In addition, evidence from peripheral ganglia studies and experimental peripheral-route transmission suggests that peripheral, cell-surface, and anatomical spread-related processes may be relevant in a strain- or subtype-dependent manner [30,31]. Emerging evidence implicating Bone Morphogenetic Protein-related signaling in prion uptake and propagation further suggests that developmental or morphogenetic processes may intersect with prion-related cellular phenotypes [32]. Beyond the established mechanisms emphasized in the prion disease literature, these findings suggest that additional, comparatively understudied aspects of prion biology may also be relevant to disease pathogenesis. In this context, the cell-surface, extracellular, adhesion-related, peripheral, developmental, and tissue-interface signatures identified here should be viewed as hypothesis-generating signals. Rather than defining definitive primary drivers of sCJD, this analysis provides a roadmap for future studies to investigate broader and less well-characterized mechanisms that may contribute to prion-associated cellular and tissue-level pathology.

The use of GO Biological Process gene sets provided a consistent enrichment framework across all molecular layers. GO-BP was selected because it offers broad disease-process coverage and could be applied uniformly to transcriptomic genes, miRNA target-derived genes, and methylation-associated genes. This consistency was important for the cross-omic comparison performed here. However, pathway and gene set resources differ in their curation rules, pathway boundaries, and gene membership, and database choice can affect enrichment results [33,34]. Therefore, the adhesion-related findings may partly reflect the structure and annotation density of the GO-BP collection.

Several additional study-design considerations should guide interpretation. Human sCJD remains rare, and available public molecular datasets are limited by sample size, tissue heterogeneity, platform differences, and variable preprocessing histories. The miRNA and DNA methylation analyses relied on internal 75/25 discovery–validation splits rather than independent external validation cohorts because only one suitable public dataset was available for each layer. These splits allowed within-layer reproducibility to be assessed, but they should not be considered equivalent to the independent transcriptomic validation performed across two separate mRNA datasets. In addition, the microarray discovery cohort and RNA-seq validation cohort were harmonized at the gene-symbol level, but the original measurement scales, feature coverage, and upstream normalization procedures differed. Therefore, shared effect-size thresholds should be interpreted as within-dataset filtering criteria rather than directly comparable quantitative effect-size measures across platforms. The use of a common per-feature testing framework also supported consistency across molecular layers, but this choice necessarily simplified layer-specific data characteristics, particularly for normalized RNA-seq values. Accordingly, differential feature results should be interpreted as screening-level signals within a hypothesis-generating comparative framework. Specifically, the use of Welch’s *t*-test for normalized RNA-seq-derived values does not model the mean–variance relationship of count-based expression data, and may therefore reduce sensitivity or bias statistical significance estimates for low-count features relative to count-aware methods such as DESeq2 or edgeR. A related limitation is that duplicate probes, transcripts, or Ensembl identifiers had to be collapsed into a single representative gene-level value for enrichment analysis. This harmonization was necessary for cross-dataset comparison, but it may miss isoform-level or transcript-specific disease signals.

The classification analyses were also exploratory and were intended to provide descriptive evidence of sample-level separation rather than validated predictive models. Since cohort sizes were small and, in some cases, imbalanced, AUC ROC estimates should be interpreted cautiously. Future predictive studies using larger independent cohorts and fuller performance reporting will be needed to evaluate the robustness and clinical relevance of these classifiers.

## 4. Materials and Methods

### 4.1. Study Design and Datasets

**Figure 7:**
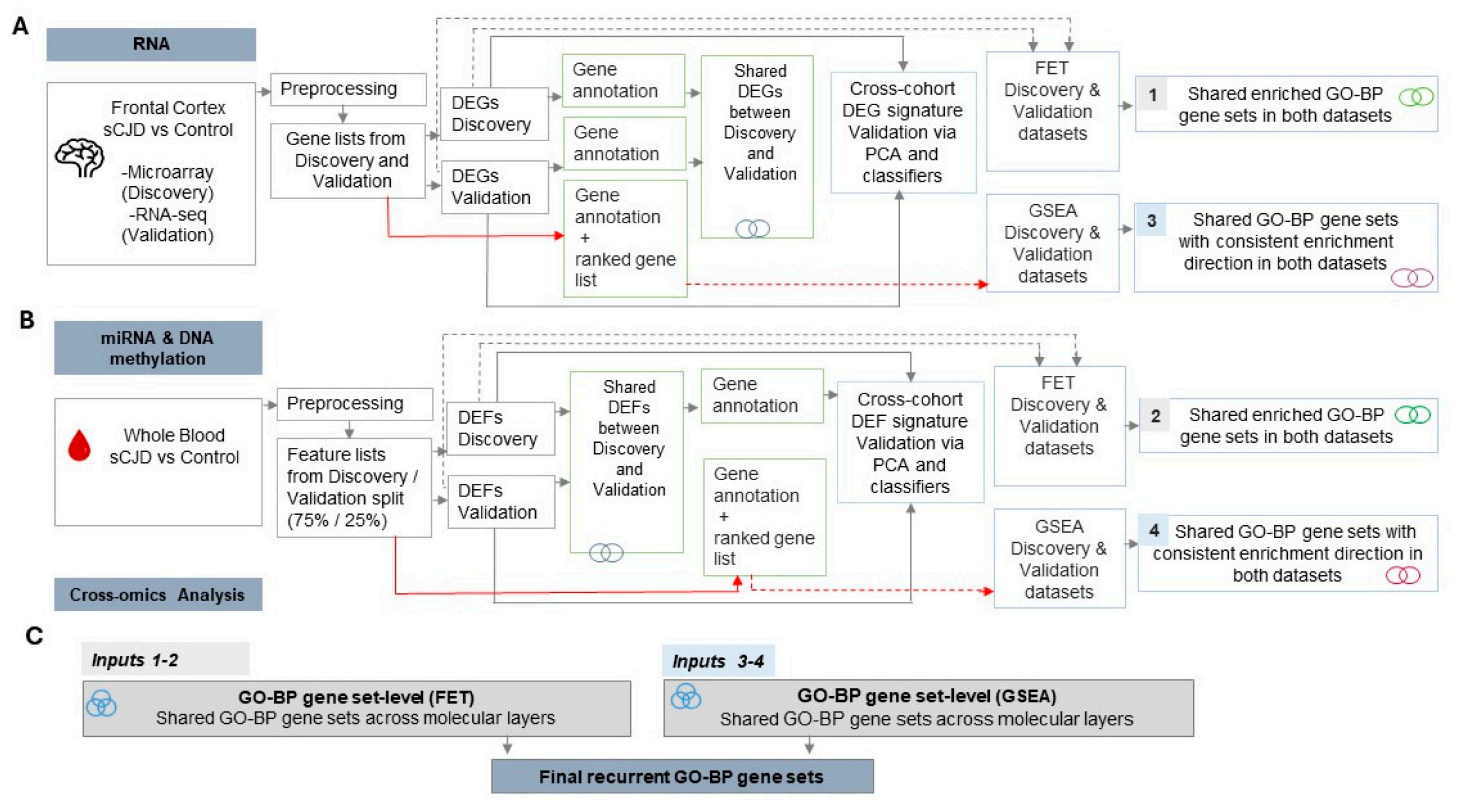
Summary of the cross-omic analysis pipeline for sCJD. Four datasets from GEO were used: two mRNA [GSE124571 (microarray) and GSE214373 (RNA-seq)], one miRNA (GSE140069), and one DNA methylation (GSE156994). (**A**) Two mRNA datasets from the frontal cortex (sCJD vs. control) were analyzed: a microarray (discovery) and an independent RNA-seq (validation) cohort, both preprocessed and log2-normalized. Differential expression analysis was performed for each dataset independently (*p* < 0.05, |log2FC| > 1, FDR < 0.05), and shared DEGs between both datasets were revealed. To assess cross-cohort discrimination, PCA was applied, followed by machine learning classifiers. Logistic regression was evaluated using leave-one-out cross-validation (LOOCV), whereas random forest was evaluated using repeated stratified k-fold validation. Classifier performance was summarized using area under the receiver operating characteristic curve (AUC ROC) and additional descriptive metrics, including sensitivity, specificity, precision, F1-score, area under the precision-recall curve (PR-AUC). Functional enrichment was performed independently for each dataset using FET and GSEA. Shared gene sets identified by FET were compared using the Jaccard index, and shared gene sets identified by GSEA were compared using normalized enrichment score (NES) correlations within each molecular layer. (**B**) The same analytical pipeline was applied to miRNA and DNA methylation datasets derived from whole blood. Since only one eligible dataset was available for each molecular layer, the miRNA and DNA methylation datasets were each internally split into discovery (75%) and validation (25%) subsets. Gene annotation for miRNA and DNA methylation was applied after identification of differentially expressed features. All other steps were performed analogously to the mRNA analysis. (**C**) Gene sets identified across molecular layers were compared to evaluate overlap at the GO-BP gene set level for both FET and GSEA results. The final recurrent GO-BP gene sets summarize exploratory nominal-threshold cross-omic gene set overlap. In the workflow diagram, gray arrows indicate the main analysis steps, red arrows indicate ranked gene-list inputs used for GSEA, dashed arrows indicate downstream transfer steps, green circles indicate shared discovery–validation features identified via FET, and purple circles indicate shared directionally consistent gene sets identified via GSEA.

Dataset characteristics are summarized in Table 6, and the relationships among preprocessing, differential analysis, validation, enrichment, and cross-omic comparison steps are illustrated in Figure 7. Publicly available datasets associated with sCJD were obtained from the NCBI Gene Expression Omnibus (GEO) and analyzed across three molecular layers: mRNA transcriptomics, miRNA expression, and DNA methylation. Four datasets were included because they contained both sCJD and control samples and could be organized within a unified discovery and validation framework. Transcriptomic profiling of post-mortem frontal cortex tissue was represented by the microarray dataset GSE124571 and the RNA-seq dataset GSE214373, which served as independent discovery and validation cohorts, respectively [17]. Whole-blood miRNA expression data were obtained from GSE140069 [18], and whole-blood DNA methylation data were obtained from GSE156994 [19]. Since only single cohorts were available for the miRNA and DNA methylation datasets, samples from these datasets were randomly partitioned into discovery and validation cohorts prior to downstream analyses.

Within each omics layer, reproducibility was evaluated at both the feature and gene set levels before performing cross-layer comparison of shared genes and gene sets. Gene set enrichment analyses were performed using Gene Ontology Biological Process (GO-BP) gene sets from the Molecular Signature Database (MSigDB) collection c5.go.bp.v2025.1.Hs.symbols.gmt [35,36]. This collection was selected because it provides broad biological-process coverage and a large, standardized framework for comparing enrichment patterns across transcriptomic, miRNA-derived target gene, and DNA methylation-associated gene lists.

### 4.2. Transcriptomic Preprocessing

For GSE124571, the GEO series matrix file was parsed to extract the probe-level expression matrix after removal of metadata rows. Values were converted to numeric format, missing or non-numeric entries were removed, and the matrix was retained on the log2 scale when appropriate according to the preprocessing configuration. Probe identifiers were mapped to gene symbols using the GPL14951 platform annotation file, and probes without usable gene annotations were excluded.

For GSE214373, depositor-provided normalized RNA-seq counts were imported from GEO. Since the exact upstream normalization procedure and RNA-seq library selection method were not fully specified in the available GEO-derived input files used for this analysis, the values were treated as normalized expression estimates and transformed as log2(count + 1) for downstream comparison. Ensembl version suffixes were removed, and features were mapped to gene symbols using the Genome Reference Consortium Human Build 38 (GRCh38) release 110 gene annotation file. When multiple Ensembl entries mapped to the same gene symbol, duplicate symbols were collapsed by taking the median expression value across samples. The resulting discovery and validation transcriptomic matrices therefore differed in their original platforms but were harmonized at the level of gene symbols for downstream comparison.

### 4.3. miRNA Preprocessing

For GSE140069, normalized miRNA abundance values were imported from GEO and transformed to the log2 scale with an offset when needed. Since only one eligible miRNA cohort was available, samples were randomly partitioned into internal discovery and validation cohorts using a 75/25 split with a fixed random seed of 42, while preserving case and control membership during sampling. Each miRNA was then linked to a representative target gene using the human microRNA Target Database (miRTarBase) interaction resource. To avoid extensive redundancy in downstream gene sets analyses, a one-to-one miRNA-to-gene mapping was generated using a greedy assignment procedure that prioritized rarer, previously unassigned target genes. Both the miRNA-level matrix and the mapped target-gene annotation table were carried forward into the differential and enrichment analyses.

### 4.4. DNA Methylation Preprocessing

For GSE156994, DNA methylation was profiled using the Illumina HumanMethylation450 BeadChip array platform (GPL13534). Therefore, the analysis was based on array-derived CpG-level β-values, with genomic coverage determined by the probes represented on the 450K array. The GEO series matrix was parsed into a CpG-by-sample β-value matrix, and samples were randomly split into internal discovery and validation cohorts using the same 75/25 design and random seed used for the miRNA dataset. To enable parametric testing, β-values were clipped at the boundaries using a small epsilon and converted to M-values using the transformation M = log2[β/(1 − β)]. M-values were used for hypothesis testing, whereas β-values were retained for effect-size estimation.

CpG probes were annotated using the GPL13534 platform file, including probe-level gene annotation for later gene-based gene set analyses. Where a probe was annotated to multiple genes, entries were expanded to enable gene-level downstream enrichment. Cross-reactive and SNP-overlapping probe filtering was not performed before differential methylation analysis. The methylation preprocessing retained probes with valid chromosome and genomic position annotations from the Illumina HumanMethylation450 platform annotation. Differentially methylated regions and regulatory annotation-level analyses, including promoter, enhancer, CpG island, and CpG shore enrichment, were not performed.

### 4.5. Differential Feature Analysis and Reproducibility Filtering

Differential analysis was performed separately in each discovery and validation cohort using Welch’s two-sample *t*-test. For transcriptomic and miRNA datasets, the effect size was defined as the difference in mean log2 expression between sCJD and control samples, reported as log2(fold change). For DNA methylation, statistical testing was performed on M-values, whereas effect size was quantified as Δβ, defined as the difference in mean β-values between sCJD and control samples. In all cases, nominal *p*-values were adjusted using the Benjamini–Hochberg procedure.

For mRNA and miRNA, differential features were summarized under both False Discovery Rate Benjamini–Hochberg (FDR-BH) ≤ 0.05 and nominal *p* < 0.05, with an additional effect-size requirement of |log2FC| > 1. Since microarray and RNA-seq expression values are not fully commensurate measurement scales, the shared |log2FC| > 1 threshold was used as a pragmatic within-dataset feature-filtering criterion to identify larger-magnitude changes within each cohort, not as a basis for directly comparing absolute effect-size magnitudes across platforms. For DNA methylation, differential CpG sites were summarized under FDR ≤ 0.05 and nominal *p* < 0.05, with |Δβ| ≥ 0.03 used as the effect-size threshold. Up-regulated and down-regulated gene lists, or hyper-methylated and hypo-methylated CpG lists, were generated separately within each cohort. Features detected in both discovery and validation cohorts with concordant direction were retained as validated signatures for downstream analyses.

### 4.6. Group-Level Validation by Dimensionality Reduction and Classification

Discovery-derived feature sets were evaluated for their ability to separate sCJD and control samples in the independent transcriptomic validation cohort or in the internal validation cohort for the miRNA and DNA methylation layers. Principal component analysis (PCA) was performed on validation matrices after z-score standardization. For supervised validation, logistic regression models were trained with standardized features and a maximum of 1000 iterations. Random forest models were trained with 500 trees, balanced class weights, and a fixed random seed of 42.

Logistic regression and random forest models were used to classify sCJD and control samples based on discovery-derived molecular signatures. Logistic regression classification was evaluated using leave-one-out cross-validation (LOOCV). Random forest classification was evaluated using repeated stratified K-fold cross-validation, with up to five folds constrained by the smaller class size and 10 repeated runs. Model performance was summarized using the area under the receiver operating characteristic curve (AUC ROC) derived from out-of-fold predicted probabilities. Sensitivity, specificity, precision, and F1-score were calculated from out-of-fold predicted class probabilities using a pre-specified probability threshold of 0.5. Precision-recall area under the curve (PR-AUC) and AUC ROC were calculated from continuous out-of-fold probabilities and were therefore threshold-independent. Since cohort sizes were small and some cohorts were imbalanced, we interpreted all classifier metrics descriptively.

### 4.7. Gene Set Enrichment Analysis

Functional enrichment was performed independently within each cohort using two complementary approaches. First, FET was used to assess the overrepresentation of validated genes within GO-BP gene sets. The GO-BP collection from MSigDB was selected as the primary enrichment resource because it provides broad biological-process coverage and the largest consistent gene set framework for comparison across transcriptomic, miRNA-derived target gene, and DNA methylation-associated gene lists. This consistency was important because the study compared heterogeneous molecular layers using both overrepresentation analysis and GSEA. The FET notebook explicitly defined a dataset-specific tested-gene universe from the full set of analyzable genes in each cohort, rather than a fixed global background. This is an important design choice because the transcriptomic, miRNA-derived, and methylation-derived feature spaces differ substantially across platforms and annotations. Second, preranked GSEA was performed with the gseapy library using genes ranked by their Welch t-statistics [37]. Where multiple entries mapped to the same gene symbol, the score with the largest absolute t-statistic was retained. GSEA analyses used 1000 permutations, minimum gene set size of 15, a maximum gene set size of 500, and a fixed seed of 123 in the notebook implementation. Results were exported as full GSEA tables and then filtered for FDR q-value < 0.05, nominal *p*-value < 0.05, or both.

### 4.8. Cross-Cohort Gene Set Reproducibility

Reproducibility of enrichment results between discovery and validation cohorts was evaluated separately for FET and GSEA. For each omics layer, overlap of enriched gene sets between cohorts was examined using Venn diagrams stratified by enrichment direction. For FET-derived gene sets, statistical significance of gene set overlap was assessed against the tested gene sets universe using FET, and the Jaccard index was calculated as a descriptive measure of set similarity. For GSEA-derived gene sets, reproducible gene sets were additionally compared by calculating Pearson and Spearman rank correlations of normalized enrichment scores (NES), which indicate the magnitude and direction of enrichment after normalization for gene set size, between cohorts, stratified by positive and negative enrichment directions. These analyses correspond to the gene set reproducibility stage of the workflow. Gene set overlap was summarized under both FDR-adjusted and nominal *p*-value thresholds.

### 4.9. Cross-Omics Comparison

Cross-omic comparison was conducted at both gene and gene set levels across transcriptomic, miRNA-derived target gene, and DNA methylation-associated results. At the gene level, validated genes from each molecular layer were compared directly to identify features shared across modalities. At the gene set level, comparisons were performed separately for results derived from FET and GSEA. Cross-omic overlap was assessed primarily using FDR-significant gene sets reproduced in both discovery and validation cohorts, with nominal *p* < 0.05 results retained as exploratory comparisons. For each modality, reproducible gene sets revealed by FET and GSEA were compiled into a unified table annotated by molecular source, within-layer regulation direction (up or down), and enrichment method. Direction labels were used as within-layer annotations to organize enrichment results and define source–direction–method contexts. These labels were not treated as directly equivalent across mRNA expression, miRNA-derived target-gene, and DNA methylation-associated gene sets. Cross-modal gene set recurrence was defined as the number of distinct source–direction–method contexts in which a gene set appeared. Recurrence scores were calculated by counting the number of unique modality × direction × method combinations associated with each gene set. To visualize relationships among recurrent gene sets, a similarity network was constructed using the 20 gene sets with the highest recurrence scores. Nodes represent gene sets, and edges represent overlap in their associated source–direction–method contexts. Pairwise similarity between gene sets was quantified using the Jaccard index. Network construction and visualization were performed using the Python library, NetworkX (version 3.6.1) [38].

### 4.10. Computational Environment and Reproducibility

All analyses were performed in Python 3.11 using nine Jupyter notebook-based workflows. Dataset preprocessing was performed using 01_prep_GSE124571_rna.ipynb, 02_prep_GSE214373_rna.ipynb, 03_prep_GSE140069_mirna.ipynb, and 04_prep_GSE156994_dna.ipynb. Gene-level analyses were performed using 05_differential_and_venn.ipynb and 06_group_level_validation.ipynb, gene set analyses using 11_FET_and_Venn.ipynb and 12_GSEA_and_Venn.ipynb, and network analysis using 13_network.ipynb.

Data handling and preprocessing used Pandas (version 3.0.1) and NumPy (version 2.4.2). Differential testing used Welch’s two-sample *t*-test from SciPy (version 1.17.0), with Benjamini–Hochberg correction implemented through statsmodels (version 0.14.6). PCA, logistic regression, random forest classification, and cross-validation used scikit-learn (version 1.8.0). Fisher’s exact test-based overrepresentation analyses used SciPy (version 1.17.0). GSEA was performed using GSEApy (version 1.1.11). Gene set similarity networks were constructed using NetworkX (version 3.6.1). Figures were generated using Matplotlib (version 3.10.8) and matplotlib-venn. Software dependencies were managed using YAML environment files to document the computational environment used for each workflow. Core dependencies were installed from 01_env.yml, while additional NetworkX dependencies required for network analysis were installed from 13_env.yml.

## 5. Conclusions

This study supports a balanced interpretation of cross-omic public-data analysis in sCJD. The strongest reproducible molecular evidence comes from transcriptomic remodeling of the affected cortex, particularly immune and glial activation alongside synaptic and neuronal suppression. Cross-layer gene-level convergence was limited, as expected given tissue and platform heterogeneity, but GO-BP gene set-level analyses identified biologically coherent hypotheses that extend beyond canonical prion disease mechanisms. In particular, adhesion- and cell-interaction-related processes emerged as exploratory signals, especially between transcriptomic and DNA methylation datasets, and may reflect broader cell-surface, extracellular, developmental, or tissue-interface biology relevant to prion disease. Rather than defining definitive primary drivers of sCJD, this analysis provides a roadmap for investigating understudied molecular and tissue-level mechanisms that may contribute to prion-associated pathology.

## Figures and Tables

**Table 1:** Gene-symbols shared between discovery and validation mRNA datasets.

Expression	Shared Gene Symbols
Over	AKR1C3, AXL, BCAS1, BTBD17, CEBPA, CEBPB, CHST6, CSF1R, CXCL16, CYBA, DDIT4, DDIT4L, DIO2, EBI3, EMP3, EPHX1, EYA2, FAM107A, FAM181A, FCGRT, FGFRL1, GNA12, GRIN2C, GYPC, HAP1, HMG20B, HSPB1, INPPL1, IRF8, ITGB2, ITGB5, ITPKB, LAPTM5, LCP1, LHX2, LRP4, LYN, MAPKAPK3, MT1X, MT2A, NACC2, NQO1, NWD1, OAF, OLR1, PARVG, PLEKHB1, PPP1R3C, PTPN6, RAB31, RAPGEF3, RENBP, RFTN2, RHBDF2, RHOQ, SIPA1, SLC25A18, SLC5A3, SLC7A2, TBL1X, TBXAS1, TGFB3, TMEM119, TNFRSF1A, TSPO, TYROBP, XAF1, and ZCCHC24
Under	ATP1A1, ATP1B1, ATP2B1, ATP6V1G2, B3GNT6, BEX1, BEX2, BEX4, BEX5, CAP2, CHGB, CLSTN3, CNTNAP2, CREG2, CRYM, CYP4X1, DCLK1, DYNC1I1, ELMOD1, ENO2, EPB41L3, FAM3C, FGF12, FGF9, GABARAPL1, GABRA1, GABRG2, GAD1, GAD2, GLRB, GLS, GNG3, GOT1, GPRASP2, HPRT1, INA, LDB2, MAL2, MAP1B, MDH1, MLLT11, MOAP1, NAP1L3, NAP1L5, NAPB, NCALD, NECAP1, NEFH, NEFM, NELL2, NMNAT2, NNAT, NSF, OPCML, PAK1, PCP4, PGM2L1, PLCXD3, PNMA2, PNMA3, PPM1E, PREPL, PVALB, RCAN2, RGS4, RIT2, RTN1, SCN3B, SLC25A12, SLC32A1, SLC38A1, SLITRK4, SNAP25, SNCA, SNCB, SST, STAT4, STEAP2, STMN1, STXBP1, SULT4A1, TAGLN3, TCEAL6, TOMM20, TSPYL1, VAMP1, VAMP2, VGF, and ZCCHC12

**Table 2:** Differentially expressed miRNA transcripts shared between discovery and validation datasets.

Direction	Shared miRNAs
Over	None
Under	hsa-miR-190a-5p (CCDC160), hsa-miR-627-3p (IFNG-AS1), hsa-miR-29a-3p (DUXAP8), hsa-miR-29c-3p (LINC00511), hsa-let-7i-5p (NEUROG1), hsa-miR-181c-5p (IL2), hsa-miR-215-5p (ANAPC15), hsa-miR-126-5p (GRIK5), hsa-miR-454-3p (MED12), hsa-miR-28-5p (LINC00265), hsa-miR-183-3p (CRNDE), hsa-miR-5695 (ABRAXAS2), hsa-miR-301a-3p (CIP2A), hsa-miR-18a-5p (CCN2), hsa-miR-93-5p (ATP6V1G2-DDX39B), hsa-miR-425-3p (PKN1), hsa-miR-33b-5p (IMPDH2), hsa-miR-4659a-5p (LYL1), hsa-miR-222-3p (CDKL5), hsa-miR-183-5p (PTPA), hsa-miR-16-5p (CHP1), hsa-miR-23b-3p (TUSC7), hsa-miR-144-3p (NT5C3A), hsa-miR-126-3p (ADGRE5), hsa-miR-19b-3p (FAM218A), hsa-miR-6513-3p (ZNF432), hsa-miR-30c-1-3p (MFSD4A), hsa-miR-342-5p (ZMYND10), hsa-miR-18b-5p (FOXN1), hsa-miR-3688-5p (DNASE1), hsa-miR-374a-5p (MIR17HG), hsa-miR-502-3p (PTF1A), hsa-miR-500a-3p (TRIM49C), hsa-miR-98-5p (ASMTL), hsa-miR-340-5p (LINC01094), hsa-miR-146a-5p (LINC00304), hsa-miR-27b-3p (LINC01234), hsa-miR-106b-5p (HOTAIRM1), hsa-miR-548k (PSMB1), hsa-miR-6866-5p (HSPA12B), hsa-miR-20a-5p (BNIP1)

**Table 3:** Differentially methylated CpGs shared between discovery and validation datasets.

Direction	Shared CpGs
Hyper-	cg22199969 (ALPI), cg19541697 (ASXL2), cg11972810 (C15orf27), cg02710296 (C1orf14), cg08787039 (C1orf14), cg04057956 (CD9), cg27396498 (CES4), cg01602287 (DUSP8), cg19369780 (DUSP8), cg14448393 (FIGN), cg21445553 (GGTLC1), cg14003931 (GPR123), cg24898738 (GPR123), cg27634264 (GPR123), cg22233843 (HLA-DQB1), cg22984282 (HLA-DQB1), cg07698121 (KIAA0182), cg14266237 (KLF1), cg04177826 (KLHDC7B), cg22249529 (KLHDC7B), cg17930194 (LHX6), cg12682972 (MAP4K4), cg21342728 (MCHR1), cg05001044 (MIR1977), cg01273330 (N/A), cg03469862 (N/A), cg03773183 (N/A), cg04160749 (N/A), cg04835511 (N/A), cg05212464 (N/A), cg05333174 (N/A), cg05740793 (N/A), cg07882059 (N/A), cg09048334 (N/A), cg10455971 (N/A), cg11166453 (N/A), cg11597902 (N/A), cg14221766 (N/A), cg14502625 (N/A), cg14538085 (N/A), cg15234197 (N/A), cg16622613 (N/A), cg17263206 (N/A), cg17598923 (N/A), cg18394854 (N/A), cg18665594 (N/A), cg21242009 (N/A), cg24330347 (N/A), cg24581226 (N/A), cg25937216 (N/A), cg26052357 (N/A), cg26684363 (N/A), cg27038634 (N/A), cg06357371 (NT5C1A), cg21929183 (PCDHGA4), cg08129505 (PGLYRP2), cg10650290 (PTPRN2), cg12955789 (RASGEF1C), cg26563141 (RGPD2), cg06039161 (SHANK2), cg13916835 (SMG6), cg07787614 (TNRC6C), cg10365886 (TNXB), cg14188106 (TNXB), cg21287054 (TTYH1), cg20543544 (ZMIZ1)
Hypo-	cg00440797 (HLA-DRB5), cg01745539 (HLA-DQB1), cg02919082 (HLA-DQA1), cg17686260 (MGMT), cg24080129 (TAP2), cg27631817 (N/A)

**Table 4:** Cross-omic gene set overlap under FDR-adjusted and nominal significance thresholds.

Analysis	FDR-Adjusted Overlap	Nominal *p* < 0.05 Overlap
FET mRNA–DNA overlap	0	18
FET mRNA–miRNA overlap	0	3
FET miRNA–DNA overlap	0	1
GSEA mRNA–DNA overlap	0	67
GSEA mRNA–miRNA overlap	0	0
GSEA miRNA–DNA overlap	0	0

**Table 5:** Shared gene sets revealed by FET in two modalities.

Modality Overlap	Gene Set	First Modality	Second Modality
mRNA and DNA	GOBP_SYNAPTIC_SIGNALING	Down	Up
	GOBP_CELL_CELL_SIGNALING	Up; Down	Up
	GOBP_CELL_ADHESION	Up	Up; Down
	GOBP_POSITIVE_REGULATION_OF_IMMUNE_RESPONSE	Up	Down
	GOBP_REGULATION_OF_CELL_ADHESION	Up	Up
	GOBP_CELL_CELL_ADHESION	Up	Up
	GOBP_GAMMA_AMINOBUTYRIC_ACID_SIGNALING_PATHWAY	Down	Up
	GOBP_GLYCINE_TRANSPORT	Down	Up
	GOBP_REGULATION_OF_GROWTH	Up	Down
	GOBP_CELL_GROWTH	Down	Down
	GOBP_AMINO_ACID_TRANSPORT	Down	Up
	GOBP_AMINE_TRANSPORT	Down	Up
	GOBP_CHLORIDE_TRANSPORT	Down	Down
	GOBP_GROWTH	Up	Down
	GOBP_INORGANIC_ANION_TRANSMEMBRANE_TRANSPORT	Down	Down
	GOBP_REGULATION_OF_AMINO_ACID_TRANSPORT	Down	Up
	GOBP_TUBE_MORPHOGENESIS	Up	Up
	GOBP_TISSUE_MORPHOGENESIS	Up	Up
MRNA and miRNA	GOBP_COGNITION	Down	Up
	GOBP_NEGATIVE_REGULATION_OF_SECRETION	Up	Up
	GOBP_REGULATION_OF_NEUROGENESIS	Up	Up
miRNA and DNA	GOBP_CALCIUM_DEPENDENT_CELL_CELL_ ADHESION_VIA_PLASMA_MEMBRANE_CELL_ ADHESION_MOLECULES	Down	Up

Note: Direction labels indicate enrichment direction within each molecular layer and were not interpreted as directly equivalent across mRNA, miRNA-derived target-gene, and DNA methylation-associated gene sets.

**Table 6:** Summary of sCJD datasets used in this study.

Dataset	Description	Platform	Probes	Annotated Probes	Unique Gene Symbols
GSE124571	mRNA samples derived from the frontal cortex of 10 sCJD patients and 10 healthy controls, and 1 duplicate (total n = 21), were analyzed by microarray.	GPL14951	24,525	24,525	18,414
GSE214373	Frontal cortex mRNA samples from 5 sCJD and 9 healthy controls (total n = 14), analyzed by RNA sequencing	GPL18573	61,906	42,072	40,861
GSE140069	Whole blood miRNA samples from 57 sCJD patients and 48 healthy controls (total n = 105), analyzed to characterize differential miRNA expression profiles.	GPL15520	939	939	939
GSE156994	Genome-wide blood DNA methylation samples from 114 sCJD patients and 105 healthy controls (total n = 219), analyzed to assess differential methylation patterns	GPL13534	403,354	307,089	19,879

## Data Availability

The datasets analyzed in this study are publicly available from repositories including the Gene Expression Omnibus (GEO). Accession numbers and dataset details are provided in Table 6.

## Supplementary Materials

The following supporting information can be downloaded at: https://www.mdpi.com/article/10.3390/ijms27156560/s1.

## Abbreviations

The following abbreviations are used in this manuscript:

AUC-ROC	Area under the receiver operating characteristic curve
BH	Benjamini–Hochberg
CJD	Creutzfeldt–Jakob disease
CpG	Cytosine–phosphate–guanine
DEG	Differentially expressed gene
DEF	Differentially expressed feature
DMR	Differentially methylated region
FDR	False discovery rate
FDR-BH	Benjamini–Hochberg false discovery rate correction
FET	Fisher’s exact test
GEO	Gene Expression Omnibus
GO-BP	Gene Ontology Biological Process
GRCh38	Genome Reference Consortium Human Build 38
GSEA	Gene set enrichment analysis
LOOCV	Leave-one-out cross-validation
MSigDB	Molecular Signatures Database
NES	Normalized enrichment score
PCA	Principal component analysis
PR-AUC	Precision–recall area under the curve
PrP	Prion protein
PrP^C	Cellular prion protein
sCJD	Sporadic Creutzfeldt–Jakob disease
SNP	Single-nucleotide polymorphism
YAML	YAML Ain’t Markup Language
