# Supplementary material for: Cross-Omic Comparative Analysis Identifies Transcriptomic Signatures and Exploratory Gene Set-Level Signals in Sporadic Creutzfeldt–Jakob Disease
Source: Int J Mol Sci. 2026 Jul 23;27(15):6560. doi: 10.3390/ijms27156560 (PMC13465255; doi:10.3390/ijms27156560)
Supplement: Supplementary file 1 [file ijms-27-06560-s001.zip › Supplemental_Figures.pdf]

Supplementary Figures

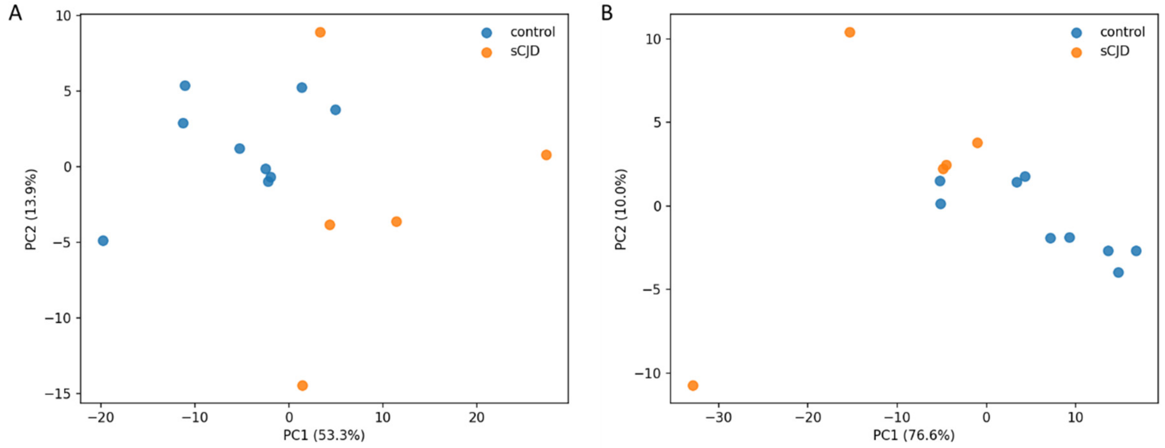

Figure S1. Principal component analysis (PCA) of transcriptomic Differentially Expressed Genes (DEG) subsets in the GSE214373 validation cohort. PCA was performed on validation-cohort expression data restricted to genes identified as differentially expressed in the discovery dataset (GSE124571). Panels show (A) over-expressed genes and (B) under-expressed genes selected at nominal $p < 0.05$. Samples are colored by phenotype (sCJD, orange; control, blue). PC1 and PC2 represent the first two principal components explaining the largest proportion of variance.

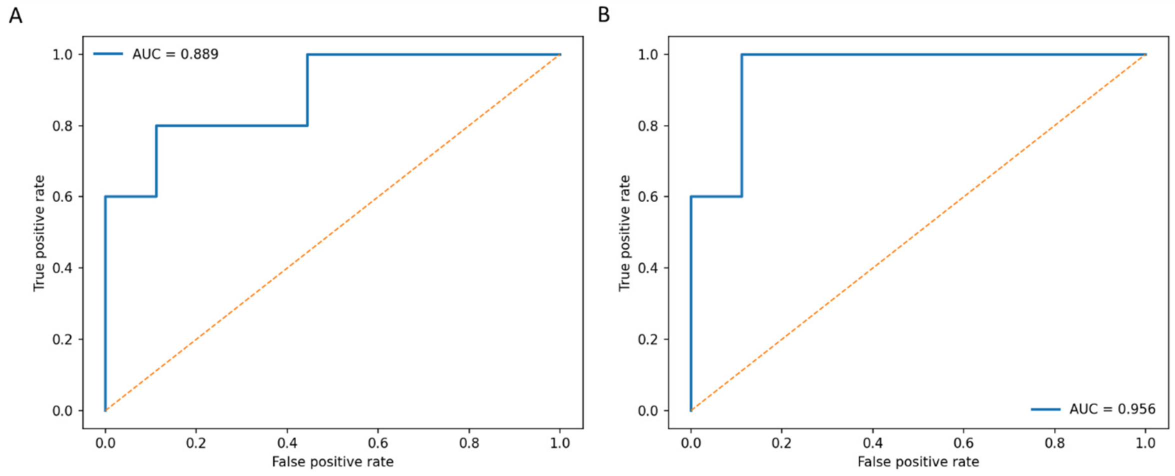

Figure S2. Logistic regression classification performance of transcriptomic DEG subsets evaluated by leave-one-out cross-validation (LOOCV) in the validation cohort (GSE214373). ROC curves are shown for gene subsets identified in the discovery dataset (GSE124571). Panels correspond to (A) over-expressed genes and (B) under-expressed genes selected at nominal $p < 0.05$. AUC values are indicated in each panel.

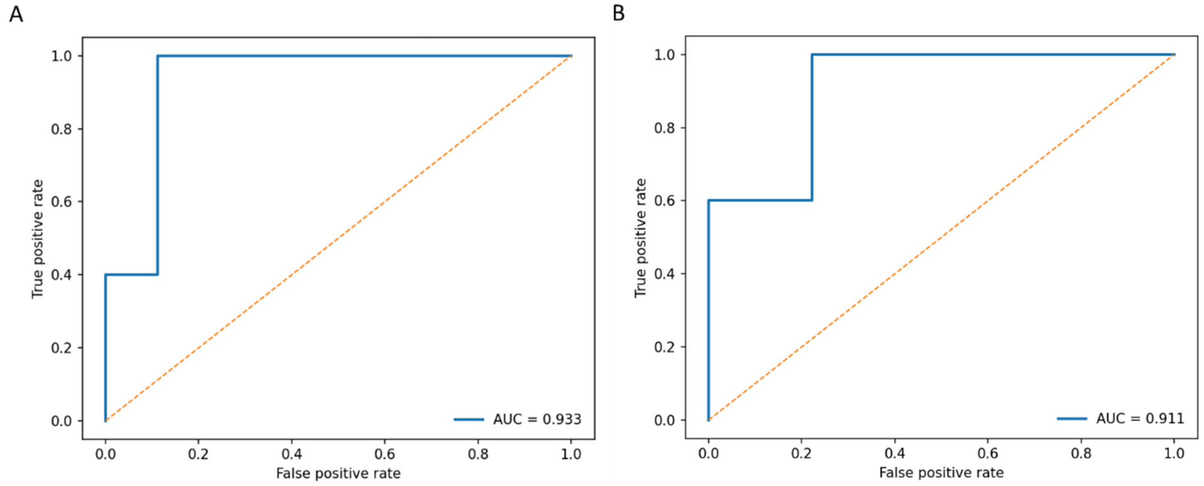

Figure S3. Random forest classification performance of transcriptomic DEG subsets in the GSE214373 RNA validation cohort. ROC curves were generated from random forest models evaluated by repeated stratified k-fold cross-validation on validation cohort expression matrices, using feature subsets selected from the GSE124571 discovery dataset. Panels show (A) over-expressed genes and (B) under-expressed genes, both selected at nominal $p < 0.05$. AUC values are indicated within each panel.

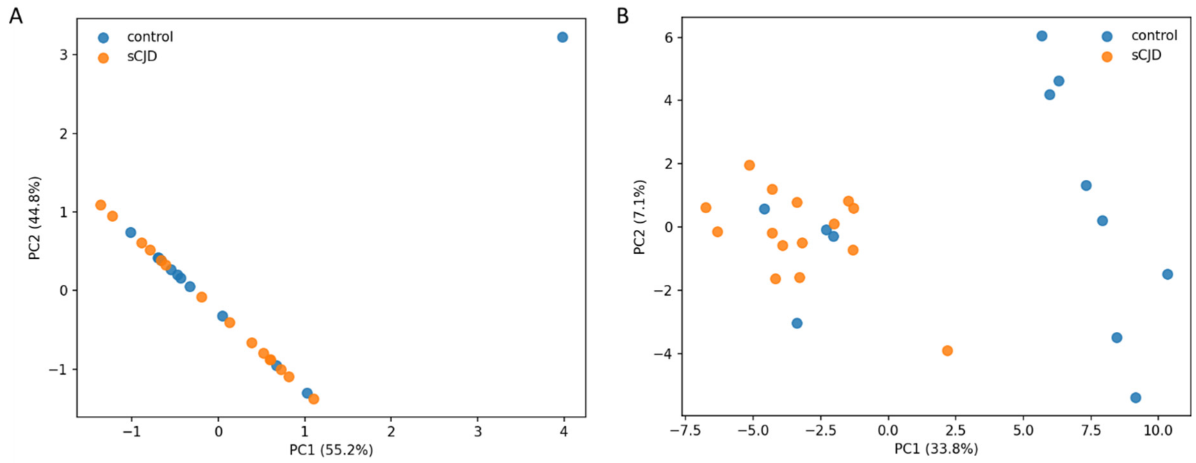

Figure S4. Principal component analysis (PCA) of miRNA subsets in the validation cohort. PCA was performed on validation-cohort expression data restricted to miRNAs identified as differentially expressed in the discovery dataset. Panels show (A) over-expressed miRNA and (B) under-expressed miRNA selected at nominal $p < 0.05$. Samples are colored by phenotype (sCJD, orange; control, blue). PC1 and PC2 represent the first two principal components explaining the largest proportion of variance. In panel A, the high combined PC1/PC2 variance reflects the very small over-expressed miRNA feature subset and should not be interpreted as robust group separation.

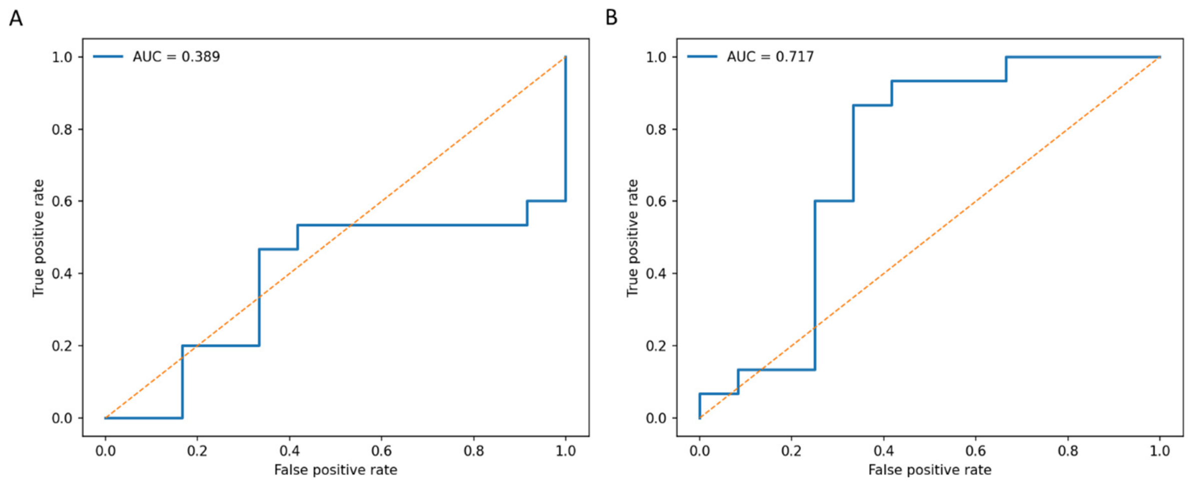

Figure S5. Logistic regression classification performance of differentially expressed miRNA subsets evaluated by leave-one-out cross-validation (LOOCV) in the validation cohort. ROC curves are shown for miRNA subsets identified in the discovery dataset. Panels correspond to (A) over-expressed miRNAs and (B) under-expressed miRNAs selected at nominal $p < 0.05$. AUC values are indicated in each panel.

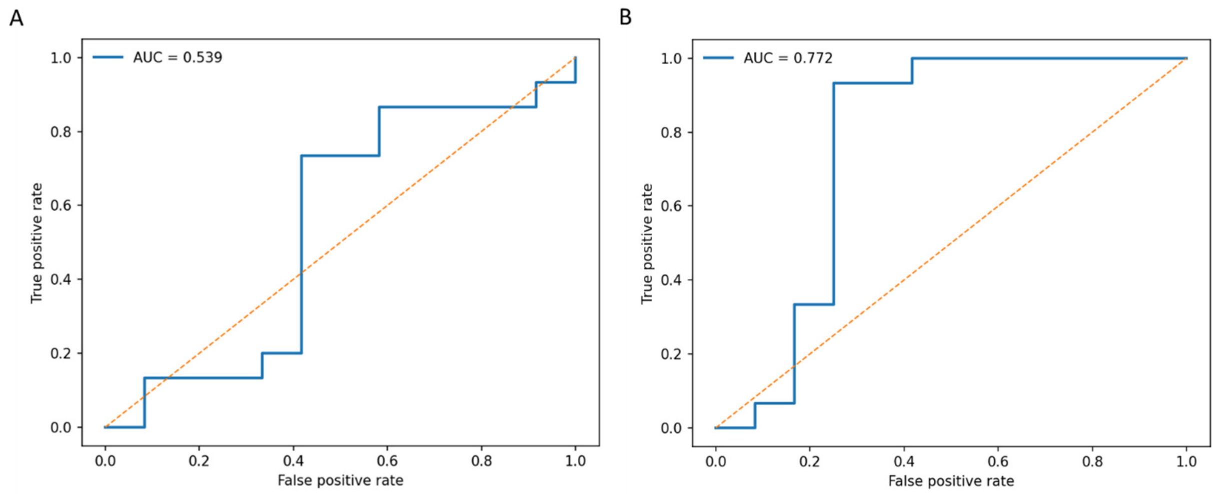

Figure S6. Random forest classification performance of differentially expressed miRNA subsets in the validation cohort. ROC curves were generated from random forest models evaluated by repeated stratified k-fold cross-validation on validation-cohort expression matrices, using feature subsets selected from the discovery dataset. Panels show (A) over-expressed miRNAs and (B) under-expressed miRNAs, both selected at nominal $p < 0.05$. AUC values are indicated within each panel.

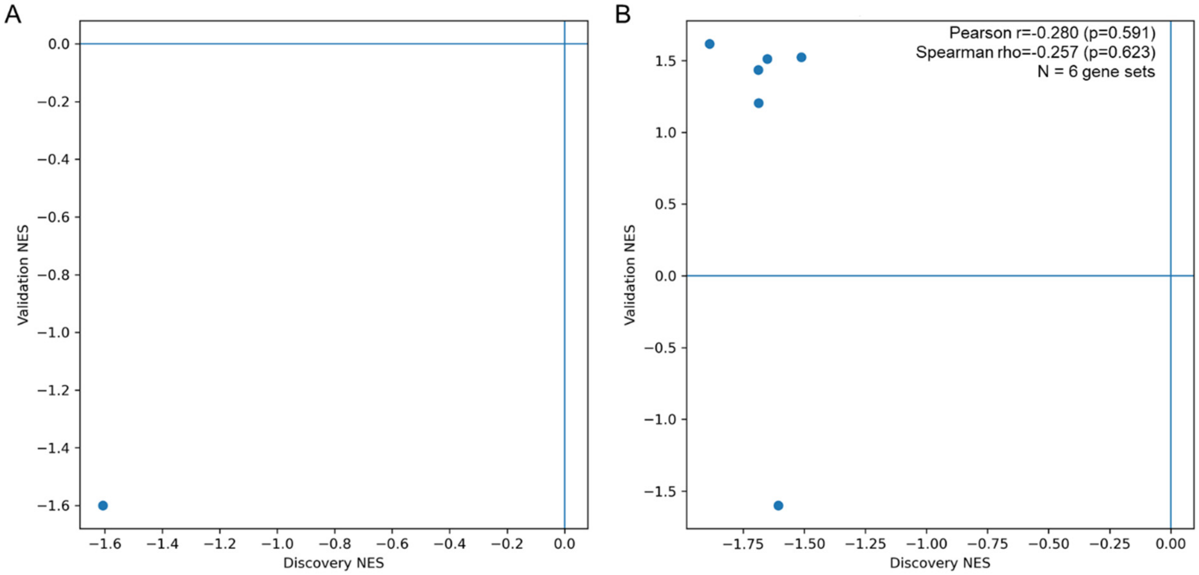

Figure S7. Concordance of GSEA normalized enrichment scores between discovery and validation cohorts for miRNA-associated gene sets. Scatter plots show normalized enrichment scores (NES) from gene set enrichment analysis comparing discovery (x-axis) and validation (y-axis) cohorts. (A) Panel A contains a single data point due to the limited number of overlapping negatively enriched pathways meeting the specified significance criteria, and therefore does not support meaningful correlation analysis. (B) Panel B displays all gene sets significant at nominal $p < 0.05$ in the discovery dataset. Each point represents a gene set. Pearson correlation (r) and Spearman rank (ρ) correlation coefficients, associated p-values, and the number of gene sets (N) are shown.

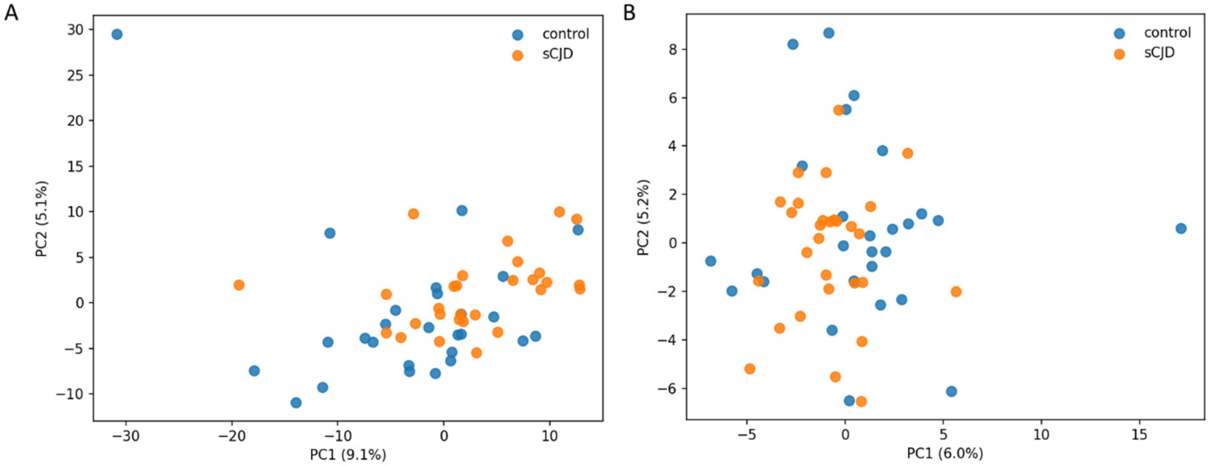

Figure S8. Principal component analysis (PCA) of CpG subsets in the GSE156994 validation cohort. PCA was performed on validation-cohort methylation data restricted to CpGs identified as differentially methylated in the discovery dataset. Panels show (A) hyper-methylated CpGs and (B) hypo-methylated CpGs selected at nominal $p < 0.05$. Samples are colored by phenotype

(sCJD, orange; control, blue). PC1 and PC2 represent the first two principal components explaining the largest proportion of variance.

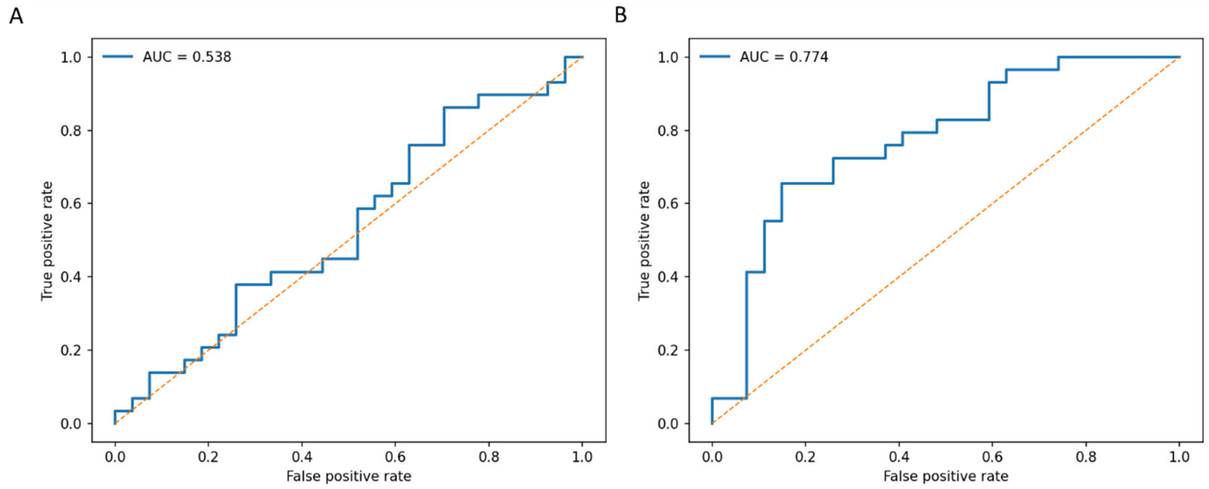

Figure S9. Logistic regression classification performance of differentially methylated CpG subsets evaluated by leave-one-out cross-validation (LOOCV) in the validation cohort. ROC curves are shown for CpG subsets identified in the discovery dataset. Panels correspond to (A) hyper-methylated CpGs and (B) hypo-methylated CpGs selected at nominal $p < 0.05$. AUC values are indicated in each panel.

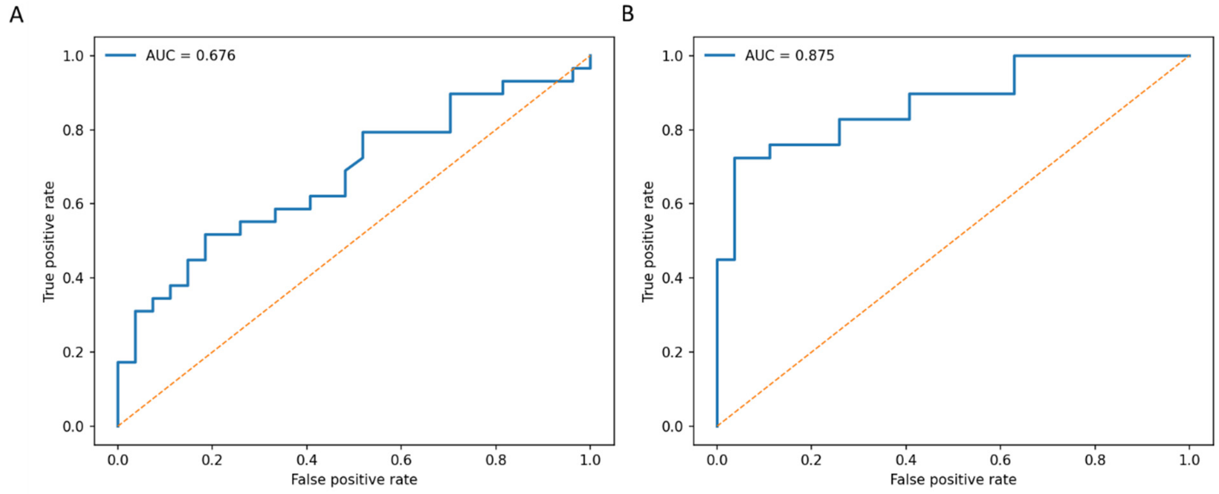

Figure S10. Random forest classification performance of differentially methylated CpGs subsets in the validation cohort. ROC curves were generated from random forest models evaluated by repeated stratified k-fold cross-validation on validation-cohort methylation matrices, using feature subsets selected from the discovery dataset. Panels show (A) hyper-methylated CpGs and (B) hypo-methylated CpGs, both selected at nominal $p < 0.05$. AUC values are indicated within each panel.

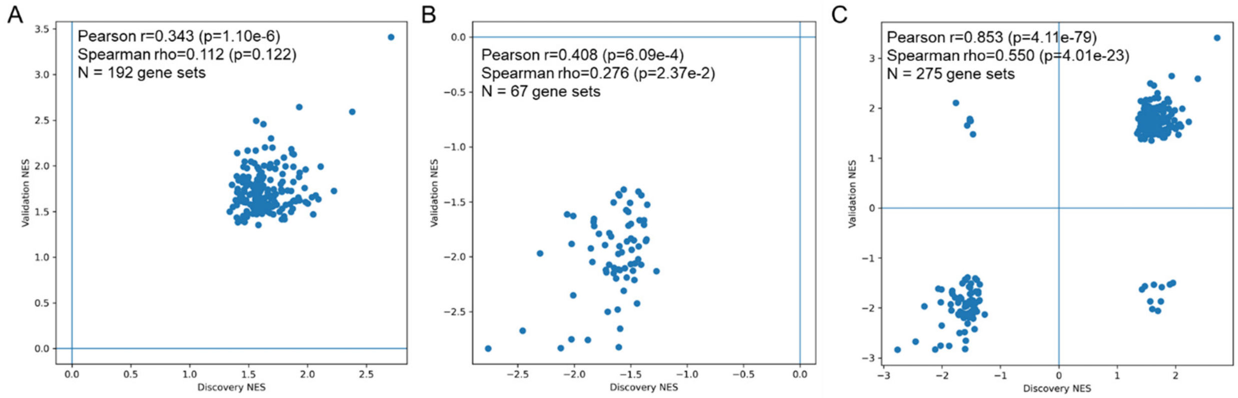

Figure S11. Concordance of GSEA normalized enrichment scores between discovery and validation cohorts for DNA methylation associated gene sets. Scatter plots show normalized enrichment scores (NES) from gene set enrichment analysis comparing discovery (x-axis) and validation (y-axis) cohorts. Panels display (A) positively enriched gene sets, (B) negatively enriched gene sets, and (C) all gene sets significant at nominal $p < 0.05$ in both discovery and validation datasets. Each point represents a gene set. Pearson correlation (r) and Spearman rank (ρ) correlation coefficients, associated p-values, and the number of gene sets (N) are shown.

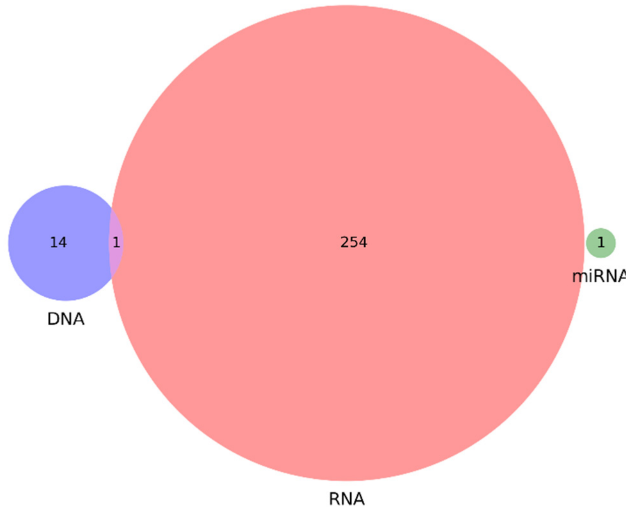

Figure S12. Gene sets supported by both FET and GSEA across omic layers.
Venn diagram showing the distribution of GO Biological Process gene sets that were identified by both overrepresentation analysis (FET) and gene set enrichment analysis (GSEA) within RNA, DNA methylation, and miRNA datasets. Gene sets were required to be nominally significant ($p < 0.05$) in both discovery and validation cohorts within each modality. A total of 270 unique gene sets were supported by both enrichment frameworks. The majority were derived from RNA data (254 gene sets), compared with 14 unique to DNA methylation and one unique to miRNA. Only one gene set was shared between RNA and DNA methylation datasets, whereas no gene sets overlapped between miRNA and the other modalities.
